# Ontology-based representation and analysis of host-*Brucella* interactions

**DOI:** 10.1186/s13326-015-0036-y

**Published:** 2015-10-05

**Authors:** Yu Lin, Zuoshuang Xiang, Yongqun He

**Affiliations:** Unit of Laboratory Animal Medicine, Department of Microbiology and Immunology, Center for Computational Medicine and Bioinformatics, and Comprehensive Cancer Center, University of Michigan Medical School, 1150 W. Medical Center Dr, Ann Arbor, MI 48109 USA

## Abstract

**Background:**

Biomedical ontologies are representations of classes of entities in the biomedical domain and how these classes are related in computer- and human-interpretable formats. Ontologies support data standardization and exchange and provide a basis for computer-assisted automated reasoning. IDOBRU is an ontology in the domain of *Brucella* and brucellosis. *Brucella* is a Gram-negative intracellular bacterium that causes brucellosis, the most common zoonotic disease in the world. In this study, IDOBRU is used as a platform to model and analyze how the hosts, especially host macrophages, interact with virulent *Brucella* strains or live attenuated *Brucella* vaccine strains. Such a study allows us to better integrate and understand intricate *Brucella* pathogenesis and host immunity mechanisms.

**Results:**

Different levels of host-*Brucella* interactions based on different host cell types and *Brucella* strains were first defined ontologically. Three important processes of virulent *Brucella* interacting with host macrophages were represented: *Brucella* entry into macrophage, intracellular trafficking, and intracellular replication. Two *Brucella* pathogenesis mechanisms were ontologically represented: *Brucella* Type IV secretion system that supports intracellular trafficking and replication, and *Brucella* erythritol metabolism that participates in *Brucella* intracellular survival and pathogenesis. The host cell death pathway is critical to the outcome of host-*Brucella* interactions. For better survival and replication, virulent *Brucella* prevents macrophage cell death. However, live attenuated *B. abortus* vaccine strain RB51 induces caspase-2-mediated proinflammatory cell death. *Brucella*-associated cell death processes are represented in IDOBRU. The gene and protein information of 432 manually annotated *Brucella* virulence factors were represented using the Ontology of Genes and Genomes (OGG) and Protein Ontology (PRO), respectively. Seven inference rules were defined to capture the knowledge of host-*Brucella* interactions and implemented in IDOBRU. Current IDOBRU includes 3611 ontology terms. SPARQL queries identified many results that are critical to the host-*Brucella* interactions. For example, out of 269 protein virulence factors related to macrophage-*Brucella* interactions, 81 are critical to *Brucella* intracellular replication inside macrophages. A SPARQL query also identified 11 biological processes important for *Brucella* virulence.

**Conclusions:**

To systematically represent and analyze fundamental host-pathogen interaction mechanisms, we provided for the first time comprehensive ontological modeling of host-pathogen interactions using *Brucella* as the pathogen model. The methods and ontology representations used in our study are generic and can be broadened to study the interactions between hosts and other pathogens.

**Electronic supplementary material:**

The online version of this article (doi:10.1186/s13326-015-0036-y) contains supplementary material, which is available to authorized users.

## Background

In the field of infectious diseases, the study of the interactive relationships between pathogens and their hosts is critically important. An infectious disease is the result of intense interactions between a pathogen and its host. During these interactions both the host and the pathogen attempt to manipulate each other using a complex network mechanism to maximize their respective survival probabilities. For the goal of survival and replication, the pathogen may adapt different pathogenesis strategies to infect the host. On the other hand, the host may apply innate and adaptive immune defense mechanisms to fight against the invading pathogen. Different live attenuated vaccines may stimulate sufficient host immunity but does not induce damaging effects on the host. For decades, scientists have conducted research to study the different aspects of host-pathogen interactions. In order to obtain a full picture of the host-pathogen interaction mechanisms, separate data from those studies needs to be integrated. Thus, a strategy of knowledge representation, management and reasoning based on the huge data resources is in need. Such a strategy will enable the knowledge integration, complicated biological data analysis, and provide insights for biologists to generate new hypotheses.

*Brucella* is a Gram-negative, non-spore-forming, facultative, intracellular bacterium that causes chronic zoonotic brucellosis in humans and a variety of animal species [1]. Human brucellosis remains the most common zoonotic disease worldwide, with more than 500,000 new human cases reported annually [2]. A safe and effective human vaccine is required but does not yet exist. A rational vaccine design would benefit from insightful understanding of the interactions between host and *Brucella*, specifically, *Brucella* pathogenesis and host defense mechanisms. *Brucella* infections are typically chronic in nature [1], suggesting a continuous interaction between host and *Brucella*. To promote its long-term intracellular survival, *Brucella* minimizes the activation of host inflammatory mechanisms. For example, *Brucella* lipopolysaccharide (LPS) has 100- to 1000-fold decreased capability to activate pro-inflammatory TNF-α and IL-1 cytokines compared to similar concentrations of *E. coli* LPS [3]. Hundreds of *Brucella* protein virulence factors participate in the *Brucella* pathogenesis and interacting with host immune systems [4, 5]. The immune systems in various *Brucella* hosts respond poorly against virulent *Brucella* strains but very well against live attenuated *Brucella* vaccine strains [6–8]. Such a complex host-pathogen interaction system involves a number of cells, molecules and biological processes. Therefore, the host-*Brucella* interactions present a good example for an ontology-based exploration of complex bacterial pathogenesis and host immunity mechanisms.

As an extension ontology of the Infectious Disease Ontology (IDO) [9], Brucellosis Ontology (IDOBRU) is developed previously at our lab [10]. Following the good practice of the OBO Foundry principles [11], IDOBRU was developed under the framework of the Basic Formal Ontology (BFO) [12] and IDO. BFO contains two branches, continuant and occurrent [11, 13]. The continuant branch represents time-independent entity such as material entity, and the occurrent branch represents time-related entity such as process. By aligning different domain ontologies under the two branches of BFO, the knowledge from broad biological areas could be captured and organized under a comprehensive ontology-level structure. Since IDO aligns with BFO, IDOBRU automatically aligns with BFO. IDOBRU exemplifies IDO in the case of brucellosis, which covers a broad range of topics, including host infection, zoonotic disease transmission, symptoms, virulence factors, pathogenesis, diagnosis, intentional release, vaccine prevention, and treatment [10].

Since the study of host-*Brucella* interactions has been a major brucellosis research effort, this paper goes beyond the simplified introduction of virulence factors and pathogenesis in the previous IDOBRU paper [10], and provides more detailed ontological representation on various aspects of the host-pathogen interactions. In this report, different types of the pathogen-side pathogenesis mechanisms and host-side immune defense strategies are described with specific examples. Comparing with the 245 virulence factors in the original IDOBRU ontology [10], current version includes 432 virulence factors in *Brucella*. Furthermore, a list of computer-understandable logic inference rules is defined in this study to make the virulence factors, host-*Brucella* interactions and related processes computable. Use cases are provided to demonstrate how such ontological representations and inference rule-based automated reasoning help data integration and query in the area of host-pathogen/vaccine interactions.

## Results

In what follows, italics are used to make ontological axioms (i.e., statements that say what is true in the domain) (http://www.w3.org/TR/owl2-syntax/), simple bold words represent ontology relations, single quotes are used to represent ontology terms, and double quotes are used for text definitions or emphases.

### Overall design of ontological representing of host-*Brucella* interaction

Various disciplines dissect interactions into different tailored meanings. The definition of an interaction in the host-pathogen interaction area includes a “two-way effect” (i.e., host’s effect upon pathogen, and pathogen’s effect upon host). The interactions between host (e.g., human) and *Brucella* exemplifies a host-pathogen interaction in the context of biology. As an intracellular bacterium, *Brucella* strains are able to invade, survive and replicate for prolonged periods within in vivo host cells or in vitro cultured host cells. The GO term ‘interspecies interaction between organisms’ (GO_0044419) is defined as “any process in which an organism has an effect on an organism of a different species”. Without capturing the granularity at the host cell level, this GO term is not sufficient for our case. The definition of ‘host-*Brucella* interaction’ in IDOBRU is “an interspecies interaction that is the physical encounter of two parties: *Brucella* and its host organism or host cell. While interacting, *Brucella* and its host organism or host cell have effects upon each other.” The interaction in this definition specifically emphasizes the physical encounter and interaction of the two parties in an action. Also, given that *Brucella* is an intracellular bacterium, it is important to explicitly mention both of the host organism and the host cell.

The host-*Brucella* interaction includes three main parts: 1) ‘*Brucella* entry into host cell’ (IDO_0101170) (the interaction at the interface between *Brucella* and host), 2) ‘process of establishing *Brucella* infection in host’ (IDO_0100426) (*Brucella* side response), and 3) ‘host anti-*Brucella* process’ (IDO_0100115) (host side response). The IDOBRU term ‘*Brucella* entry into host cell’ is a child term of GO ‘entry of bacterium into host cell’ (GO_0035635). The IDOBRU term ‘process of establishing *Brucella* infection in host’ is a child term of the IDO-core term ‘process of establishing an infection’ (IDO_0000603), which is under GO ‘biological process’. The ‘host anti-*Brucella* process’ is a child term of GO ‘biological process’.

As we mentioned before, IDOBRU adopted Basic Formal Ontology version 2 (BFO 2) as its top level ontology [10]. Favoring BFO is due to the integrative nature of IDOBRU, a representation of all aspects of brucellosis, as it requires integrating with other OBO library ontologies, including the Cell Type Ontology (CL) [14], Chemical Entities of Biological Interest (ChEBI) [15], Gene Ontology (GO) [16], Information Artifact Ontology (IAO) [17], Ontology of Biomedical Investigations [18], Ontology of General Medical Science (OGMS) (https://code.google.com/p/ogms/), Ontology of Genes and Genomes (OGG) [19], and Protein Ontology (PRO) [20]. BFO serves as a common structure and a formal framework to seamlessly integrate existing terms from all OBO foundry ontologies. Under this consideration, we adopt relations formally defined by the community as much as possible. To represent the relations between molecular entities and its related host-*Brucella* interactions, we adopted the relation **has_agent** formally defined by Smith et al. [21]. In the definition provided by Smith et al., a *p****has_agent*** c ***at*** t denotes a primitive relation between a process *p*, a continuant *c* and a time *t* at which the continuant *c* is causally active in the process [21]. The notion of “causally active” is aimed to capture the “from-to” directionality nature of a biological process, which provides the explicit measure for the “two-way effect” interactions. Applying this relation to model host-*Brucella* interaction, we differentiate the host-*Brucella* interaction into two categories: 1) ‘*Brucella* process towards host infection’, and 2) host anti-*Brucella* process. The first category has *Brucella* as its agent, and the second category has a host as its agent. In another word, *Brucella* actively causes ‘establishing *Brucella* infection in host’, and host actively causes host anti-*Brucella* processes. The *Brucella* and host both play an agent role in the processes of host-*Brucella* interaction.

To go beyond the textual definitions and logically define the ‘host-*Brucella* interaction’ and many other ontology terms, two approaches were used. One approach was to use an ontological axiom(s). An ontological axiom is a statement that provides explicit logical assertions about three types of things: classes, individuals and properties (http://www.w3.org/TR/owl2-syntax/#Axioms). The other facts implicitly contained in the ontology can be inferred using a reasoning software program (i.e., a reasoner). Another approach is based on inference rules. On the Semantic Web, the term “inference” means an automatic procedure that can generate new relationship(s) based on the data (e.g., ontology knowledge data) and some additional information in the form of a vocabulary, for example, a set of rules (http://www.w3.org/standards/semanticweb/inference). An inference rule (IR) is a logical form consisting of a function that takes premises, analyzes the syntax, and returns a conclusion(s).

To improve data integration and discover new relationships and possible inconsistencies, we have defined seven inference rules (IRs) in this report. We have first developed inference rules to capture the physical interaction of the two entities (agents) in a host-*Brucella* interaction. We use the IF *p* THEN *q* inference rules to state that, given the truth of *p*, allows the truth of *q* to be inferred. The IF…THEN rules are used as a knowledge representation format that can be easily understood and simple to implement by a computer [22]. Here we formalized the first inference rule (IR1) to define a host-*Brucella* interaction as following:

(**IR1**) IF (*a***agent_in***p,* ∩ *b***agent_in***p*)*,* ∩ *p***is_a** process*,* ∩ (*a***part_of***A*, ∩ *b***part_of***B*) ∩ (*A***is_a** (host organism ∪ host cell)*,* ∩ *B***is_a***Brucella*), THEN *p***is_a** ‘host-*Brucella* interaction’

IR1 gives three constrains sufficient to define a direct host-*Brucella* interaction: 1) two entities *a* and *b* are agents in a process; 2) these two entities are parts of entity *A* and *B* respectively; 3) *A* is a host organism or host cell, and *B* is a *Brucella* bacterium. *A* and *B* are disjoint with each other. It is noted that while a direct host-*Brucella* interaction involves both host and *Brucella* molecules, a process that participates in the host-pathogen interaction may be just a typical host or pathogen process that is triggered by the direct host-*Brucella* interaction. In this article, we will provide many examples showing the pathogen-side or host-side processes following a direct host-*Brucella* interaction.

Besides IR1, this article will provide six other IF…THEN inference rules. The IR2 provides an inference that one process is preceded by another process after a direct host-*Brucella* interaction. IR3-IR7 is about the inference on a virulence factor. Different from ontology axioms that usually represent necessary or (necessary and sufficient) conditions, these IF…THEN inference rules represent sufficient criteria (IF conditions) for a specific inference (THEN conclusion).

While ontology axioms behave like inference rules, the Web Ontology Language (OWL) is unable to express all relations and inference rules (http://dior.ics.muni.cz/~makub/owl/#ontology). One common language that can be used to define inference rules is the Semantic Web Rule Language (SWRL) developed based on a combination of the OWL language with the Rule Markup Language (http://www.w3.org/Submission/SWRL/). While inference rules do not have to be a part of the ontology, SWRL can be used to represent the rules in an ontology like IDOBRU as shown in Additional file 1. These inference rules described in an ontology have at least two functions. First, these rules in combination with a logic reasoner support ontology consistency check. If an ontology has errors in conflict with an inference rule, a reasoner like Hermit (http://hermit-reasoner.com/) will be able to detect the error. Second, an IF…THEN inference rule can generate a conclusion on an ontology instance based on specified conditions.

While inference rules are less frequently used in biomedical ontologies, the SPARQL Protocol and RDF Query Language (SPARQL) has been widely used for querying ontologies [23] and is familiar to the general readers in biomedical semantics. In this article, we have also provided many SPARQL examples to illustrate the usage of SPARQL in querying information related in host-*Brucella* interactions.

### Ontology modeling of different types of *Brucella*

*Brucella* strains can be separated into smooth strains and rough *Brucella* strains depending on their lipopolysaccharide (LPS) compositions. As a major component of the outer membrane of *Brucella*, *Brucella* LPS is composed of three parts: O polysaccharide (or called O-chain or O-antigen), core oligosaccharide, and lipid A. The O polysaccharide, a repetitive glycan polymer attached to the core oligosaccharide, is the outermost domain of the LPS molecule. The presence or absence of the LPS O polysaccharide determines whether a *Brucella* strain has the smooth or rough phenotype, respectively. The presence of full-length O polysaccharides would render the bacterium smooth, whereas the absence of O polysaccharides would make the bacterium rough [24]. Rough *Brucella* is usually attenuated, and it does not stimulate the production of anti-O polysaccharide antibody in an infected host. In contrast, most smooth *Brucella* strains are virulent, and they have intact O polysaccharides and can stimulate anti-O polysaccharide antibody in host [25]. Virulent wild type *B. abortus*, *B. melitensis*, *B. neotomae*, and *B. suis* are all smooth strains, and their rough strains (including many vaccine and vaccine candidate strains) are attenuated. However, virulent wild type *B. canis* and *B. ovis* are rough. Therefore, the virulent/attenuated and smooth/rough characteristics of *Brucella* strains may not match each other.

In order to define the smooth and rough characteristics of *Brucella,* the negative statement of ‘has no *Brucella* O polysaccharide’ needs to be addressed. Ceusters et al. has given a set of ‘lacks’ relations to represent the negative findings in electronic health records [26]. For example, the relation **lacks_part** was defined in terms of the positive relation **part_of**, holds between a particular *p* and a universal *u* when *p* has no *u* as part [26]. However, all relations in OWL are relations between particulars by default and cannot represent the relation between a particular and a universal. It is possible to rely on the punning (an OWL2 feature) that allows OWL developers to use the URI of a class for an individual (http://www.w3.org/TR/owl2-new-features/#F12:_Punning). Another shortcoming about the **lacks_part** relation in OWL is that the relation in OWL expression *C subClassOf:***lacks-part***some D* implies the presence of an instance of D where the relation itself suggests the lack of such an instance [27]. Therefore, although initially we used the **lacks_part** relation, we have recently switched to the use of **not has_part** as suggested in the article [27]. Here we adopt their strategy to define the terms ‘smooth *Brucella* strain’ and ‘rough *Brucella* strain’ as follows:

*Smooth Brucella strain =*_*def*_*a Brucella has_part some ‘smooth Brucella lipopolysaccharide’*

*Rough Brucella strain =*_*def*_*a Brucella has_part only ‘rough Brucella lipopolysaccharide’*

*Smooth Brucella lipopolysaccharide =*_*def*_*a ‘Brucella lipopolysaccharide’ has_part some ‘Brucella O polysaccharide’*

*Rough Brucella lipopolysaccharide =*_*def*_*a ‘Brucella lipopolysaccharide’ not has_part some ‘Brucella O polysaccharide’*

### Ontology modeling of host-*Brucella* interaction subtypes

*Brucella* bacteria are able to invade and infect both professional and non-professional phagocytes. The interactions between *Brucella* and these host cells dictate the outcomes of the infection [28]. At least three types of host cells are recognized: macrophages, dendritic cells, and epithelial cells (e.g., placental trophoblast cells) [28]. Some cell lines, such as mouse macrophage cell line J774 [25] and human epithelial cell line HeLa [29] are important models for studying host-*Brucella* interactions. Those cell types and cell lines have been imported into IDOBRU from Cell Type Ontology (CL) [14] and Cell Line Ontology (CLO) [30] respectively by using OntoFox, a web-based tool for retrieving and extracting ontological terms and axioms [31].

Given the above three types of host cells and two types of *Brucella* strains, we have asserted several subtypes of host-*Brucella* interactions using the format of ‘*host cell – Brucella interaction’*. Specifically, six subtypes of host-*Brucella* interactions were asserted:

1. macrophage – smooth *Brucella* interaction
2. macrophage – rough *Brucella* interaction
3. dendritic cell – smooth *Brucella* interaction
4. dendritic cell – rough *Brucella* interaction
5. epithelial cell – smooth *Brucella* interaction
6. epithelial cell – rough *Brucella* interaction.

Different agents participate in each of the host-*Brucella* interactions. For example, the agents in macrophage-*Brucella* interactions include:

1. *‘Brucella’***agent in***‘macrophage – Brucella interaction’*
2. *‘Brucella’***agent in***‘process of establishing Brucella infection in macrophage’*
3. *(‘macrophage’ or ‘macrophage cell line cell’)***agent in***‘macrophage – Brucella interaction’*
4. *(‘macrophage’ or ‘macrophage cell line cell’)***agent in***‘macrophage anti-Brucella process’*
5. *‘macrophage-Brucella interaction’***has_part***‘process of establishing Brucella infection in macrophage’*
6. *‘macrophage-Brucella interaction’***has_part***‘macrophage anti-Brucella process’*

**Fig. 1:**
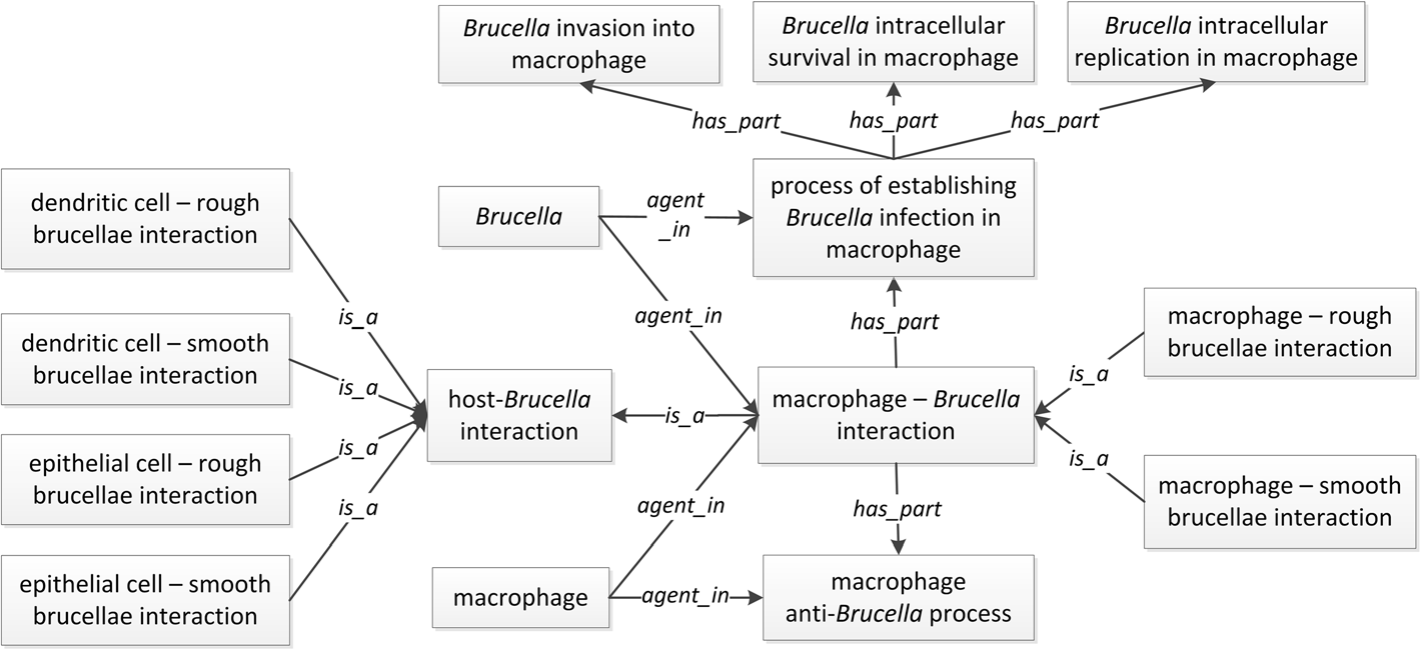
Ontological representation of various interactions between *Brucella* and host cells

Figure 1 illustrates the above triples and gives six subtypes of macrophage-*Brucella* interactions.

It is noted that the above categorizations do not count on the interactions between *Brucella* and host organ (e.g., spleen) or the whole host at an organism level. As *Brucella* is an intracellular bacterium, the macrophage-*Brucella* interaction is critical to the outcome of the host-*Brucella* interaction [32]. In this article, we will primarily use the macrophage-*Brucella* interaction processes as an example for modeling the host-*Brucella* interactions.

### Ontology representation of *Brucella* invasion, trafficking, and replication inside host cells

As an intracellular bacterium, the invasion, survival and replication of *Brucella* inside host cells are crucial to *Brucella’s* lifecycle. The ultimate goal of *Brucella* is to propagate in their preferred niche in host cells (particularly the macrophages), where they can reach extensive replication and subsequently transmitted to new host cells. The intracellular life of *Brucella* is a subject of intensive scientific research [28, 32].

Specifically, through different modes of entry into a macrophage (details given in the following section), a smooth or rough *Brucella* cell will enter a *Brucella-*containing vacuole (BCV) inside a macrophage. The BCVs containing smooth and rough *Brucella* cells undergo different intracellular trafficking pathways. Smooth BCVs become mature replicative niche, where the bacteria undergo extensive intracellular replications. In such a replicative niche, programmed macrophage cell death is prevented, which is beneficial for the intracellular *Brucella*. In contrast, rough bruellae are fused with lysosome and cannot replicate inside macrophages [28]. The rough BCV will undergo programmed cell death [25, 33–35]. The process of rough *Brucella* interacting with macrophages precedes the process of programmed macrophage cell death, which is beneficial for the host.

#### *Brucella*: from entry into host cell to replication niche

**Fig. 2:**
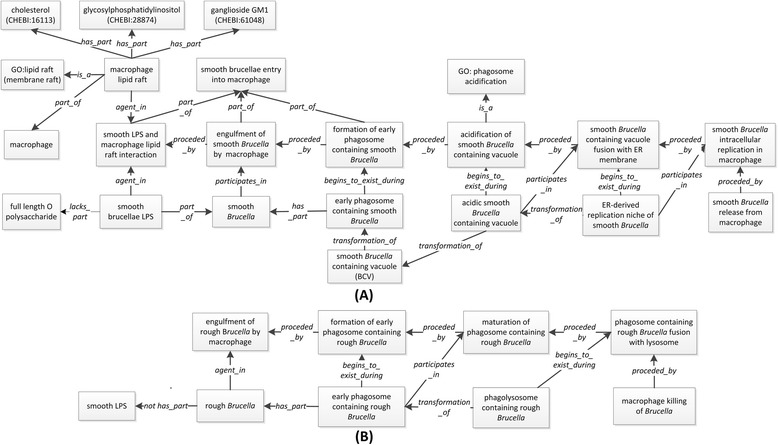
Ontological representation of the entrance into macrophage to replication by smooth *Brucella* (**a**) and rough *Brucella* (**b**)

Smooth and rough *Brucella* strains utilize different mechanisms of entry into host cells. The *Brucella* LPS O polysaccharide is a critical molecule for interaction with lipid rafts within the plasma membrane of a host cell. The lipid rafts mediate the internalization of *Brucella* by phagocytes and nonprofessional phagocytes in a manner that leads to the development of the replicative niche [28]. The ontological representation of *Brucella* entry into macrophages and other related processes is shown in Fig. 2.

As shown in Fig. 2a, a smooth *Brucella* LPS (part of *Brucella*) and a lipid raft from a macrophage (part of macrophage) are both agents in the process of the interaction between smooth LPS and macrophage lipid raft. A lipid raft is composed of cholesterol (CHEBI_16113), ganglioside GM1 (CHEBI_61048), and glycosylphosphatidylinositol (CHEBI_24410). The LPS-lipid raft interaction leads to the engulfment of smooth *Brucella* into a macrophage, preceded with the formation of an early phagosome containing smooth *Brucella*. This early phagosome does not fuse with late endosome or lysosome [28]. The smooth *Brucella* containing vacuole (BCV) is acidified and trafficked to an endoplasmic reticulum (ER), where the BCV is fused with ER membrane. The fusion event leads to the formation of ER-derived replication niche of smooth *Brucella*, where the intracellular replication takes place. It is noted that only a small percentage of all invading *Brucella* cells will survive and achieve their goal of intracellular replications through the trafficking pathway.

Compared to smooth *Brucella*, rough *Brucella* cells have a different fate (Fig. 2b). Since rough *Brucella* does not have LPS O polysaccharide, rough *Brucella* enters into a macrophage via direct macrophage engulfment rather than the lipid raft-dependent engulfment [36]. The early rough *Brucella-*containing phagosome is fused with lysosome to form a rough *Brucella*-containing phagolysosome, where the invading bacterium is killed by the macrophage [36].

To formalize the representation of *Brucella* intracellular trafficking pathways, five formal relations defined in BFO were used: **participates in**, **initially participates in**, **transformation of**, **begins to exist during,** and **starts_during**. The terms **participates_in** and its subclasses **initially participates in** and **begins_to_exist_during** are three relations between continuant and process. If a continuant *c***begins to exist during** a process *p*, it infers that continuant *c* does not exist before *p* starts. If a continuant *c***initially participates in** a process *p*, it infers that *p* cannot start without the existence of continuant *c*. **Transformation_of** links continuants in a similar fashion as preceded_by linking processes. Specifically, if continuant *c1***transformation_of** continuant *c2*, it infers that *c2* exists earlier than *c1*. The term **starts_during** represents a relation between two processes. Taken these five relations together, the following IR2 is developed to infer the temporal relations between two processes:

(**IR2**) IF *c1***participates in***p1*, ∩ *c***initiatively participates in***p2*, ∩ *c2***begins_to_exist_during***p1*, ∩ *c2***transformation_of***c1*, THEN *p2***starts_during***p1*

For example, based on the biological fact that an early smooth *Brucella*-containing phagosome is transformed to smooth *Brucella*-containing vacuole (Fig. 2), the following statements are represented in IDOBRU:

1. *‘acidic phagosome containing smooth Brucella’***participates in***‘smooth Brucella containing phagosome fusing with ER membrane’*
2. *‘ER-derived replication niche of smooth Brucella’***initiatively participates in***‘smooth Brucella intracellular replication in macrophage’*
3. *‘ER-derived replication niche of smooth Brucella’***begins_to_exist_during***‘smooth Brucella containing phagosome fusing with ER membrane’*
4. *‘ER-derived replication niche of smooth Brucella’***transformation_of***‘acidic phagosome containing smooth Brucella’*

Based on IR2, it is inferred that *‘smooth Brucella intracellular replication in macrophage’****starts_during****‘smooth Brucella containing phagosome fusing with ER membrane’*. The inferred statement is biologically valid [6].

As described in the Methods section, IR2 has been added to IDOBRU using the SWRL syntax (Additional file 1).

#### Representing intracellular survival of smooth Brucella inside macrophages

While a smooth *Brucella*-containing vacuole is trafficking within a host cell, the bacteria inside the vacuole encounter formidable environmental stresses such as the exposures to reactive oxygen (ROS) and nitrogen species (RNS), acidic pH, nutritional deprivation, and lytic peptides contained in lysosomes [28]. To withstand these environmental stresses, *Brucella* has developed different strategies.

IDOBRU uses several ‘smooth *Brucella* resistance’ subclass terms to represent the resistant processes enabling smooth *Brucella* to survive under the stressful environments within macrophages. Examples of such terms are: ‘smooth *Brucella* resistance to nutrient deprivation’, ‘smooth *Brucella* resistance to antimicrobial peptide’, ‘smooth *Brucella* resistance to nitrosative stress inside BCV’, ‘smooth *Brucella* resistance to oxidative stress inside BCV’, and ‘smooth *Brucella* resistance to acidity stress inside BCV’. The ‘part of’ relation was used to link above processes to ‘process of establishing smooth *Brucella* survival in macrophage’ in the ontology.

**Fig. 3:**
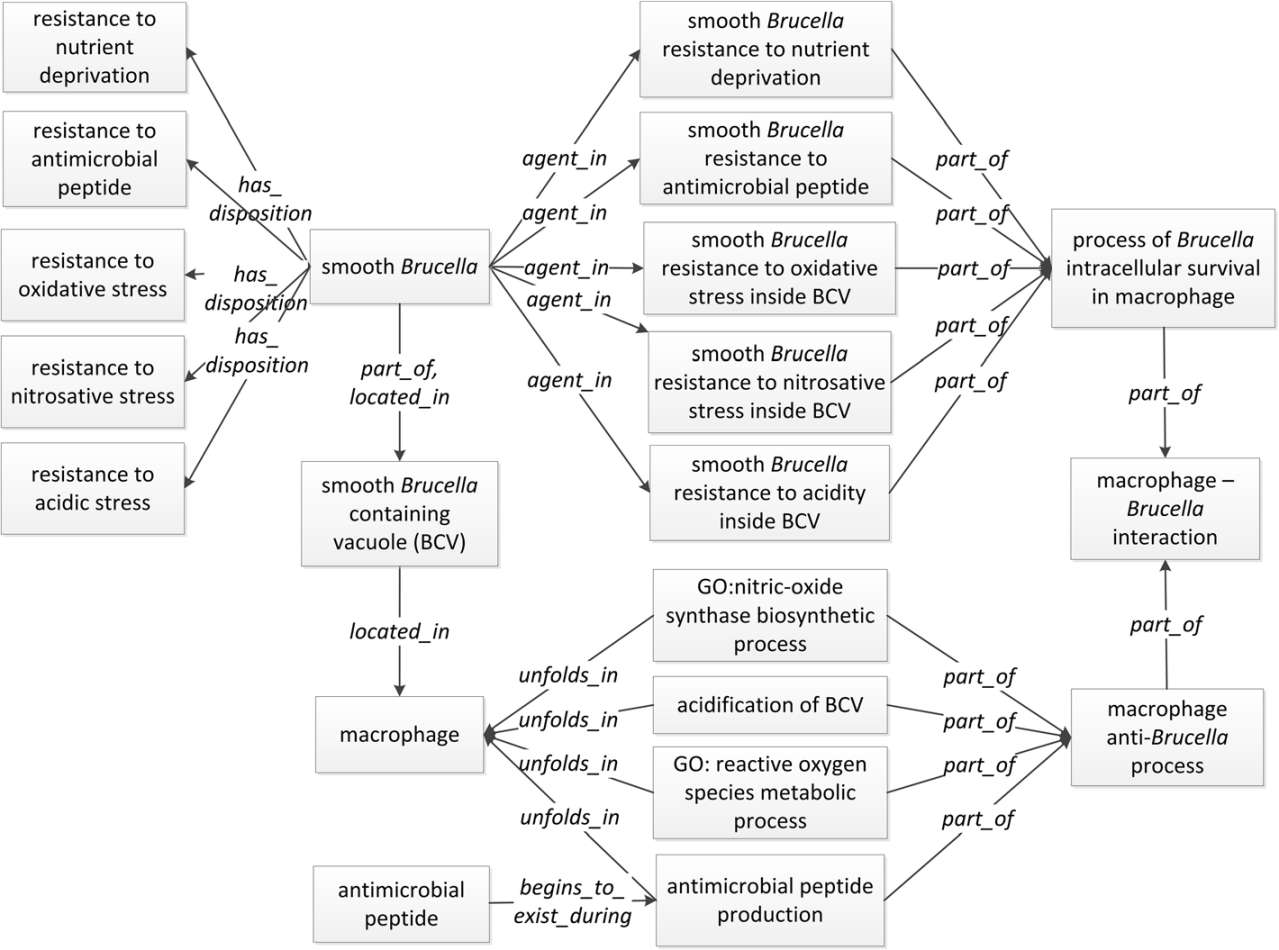
Ontological representation of *Brucella* intracellular survival inside macrophages

The abilities of smooth *Brucella* resistance to those stressful conditions were represented as different types of ‘disposition’. The IDO term ‘protective resistance’ is used as their direct mother term in virtue of protecting *Brucella* from different stresses. Smooth *Brucella* has the dispositions including ‘nutrient deprivation resistance disposition’, ‘antimicrobial peptide resistance disposition’, ‘oxidative stress resistance disposition’, ‘nitrosative stress resistance disposition’, and ‘acidic stress resistance disposition’. These dispositions are realized in relevant resistance processes and inhere in a ‘smooth *Brucella* strain’. For example, the ‘nutrient deprivation resistance disposition’ of smooth *Brucella* is realized in the process of ‘smooth *Brucella* resistance to nutrient deprivation’, and ‘smooth *Brucella* strain’ is the agent participating in the process (Fig. 3).

As the interactions are between host and pathogen, the response actions of macrophage cells are represented in IDOBRU using four biological process terms: ‘nitric-oxide synthase biosynthetic process’ (GO_0051767), ‘reactive oxygen species metabolic process’ (GO_0072593), ‘acidification of BCV in macrophage’ (IDO_0100758), and ‘macrophage antimicrobial peptide production’ (IDO_0100759). All these processes are part of the ‘macrophage anti-*Brucella* process’. The **unfolds_in** relation is used to capture the fact that those processes take place inside a macrophage cell (Fig. 3).

### Representing *Brucella* pathogenesis mechanisms

In macrophages, the majority of BCVs fuse with lysosomes and the bacteria are killed and degraded in the early hours of internalization [36]. Approximately 10–30 % of internalized *Brucella* cells are able to survive and undergo the intracellular trafficking of the host cell [37]. The molecular mechanisms of *Brucella* pathogenesis are responsible for all kinds of interactions with their mammalian hosts. Virulent *Brucella* employs several strategies and uses many virulence factors to establish and maintain persistent intracellular residence in host cells. Intracellular *Brucella* also alters biological functions of professional phagocytes, resulting in the losing of their robust antigen-processing capacity. In order to prevent more hostile extracellular environment, virulence *Brucella* is able to prevent the programmed cell death of infected macrophages [38]. *Brucella* pathogenesis relies on the presence of many *Brucella* virulence factors and their interactions with the host defense system. Two examples are generated here to illustrate how IDOBRU represents the interactions between host and bacterial genes, proteins and pathways.

#### Representing type IV secretion system

Bacterial type IV secretion systems (T4SS) are often critical to selective translocation of proteins and DNA–protein complexes [39]. *Brucella* T4SS, encoded by the *virB* operon, is a major virulence factor system [38–41]. *Brucella* T4SS *virB* operon includes 12 genes whose expression is specifically induced by the phagosome acidification after *Brucella* phagocytosis [40]. *Brucella* T4SS is required for smooth *Brucella* trafficking to replication niches and intracellular survival inside host cells. In rough strain of *Brucella*, T4SS expression is important for *Brucella* cytotoxicity in macrophages [38].

**Fig. 4:**
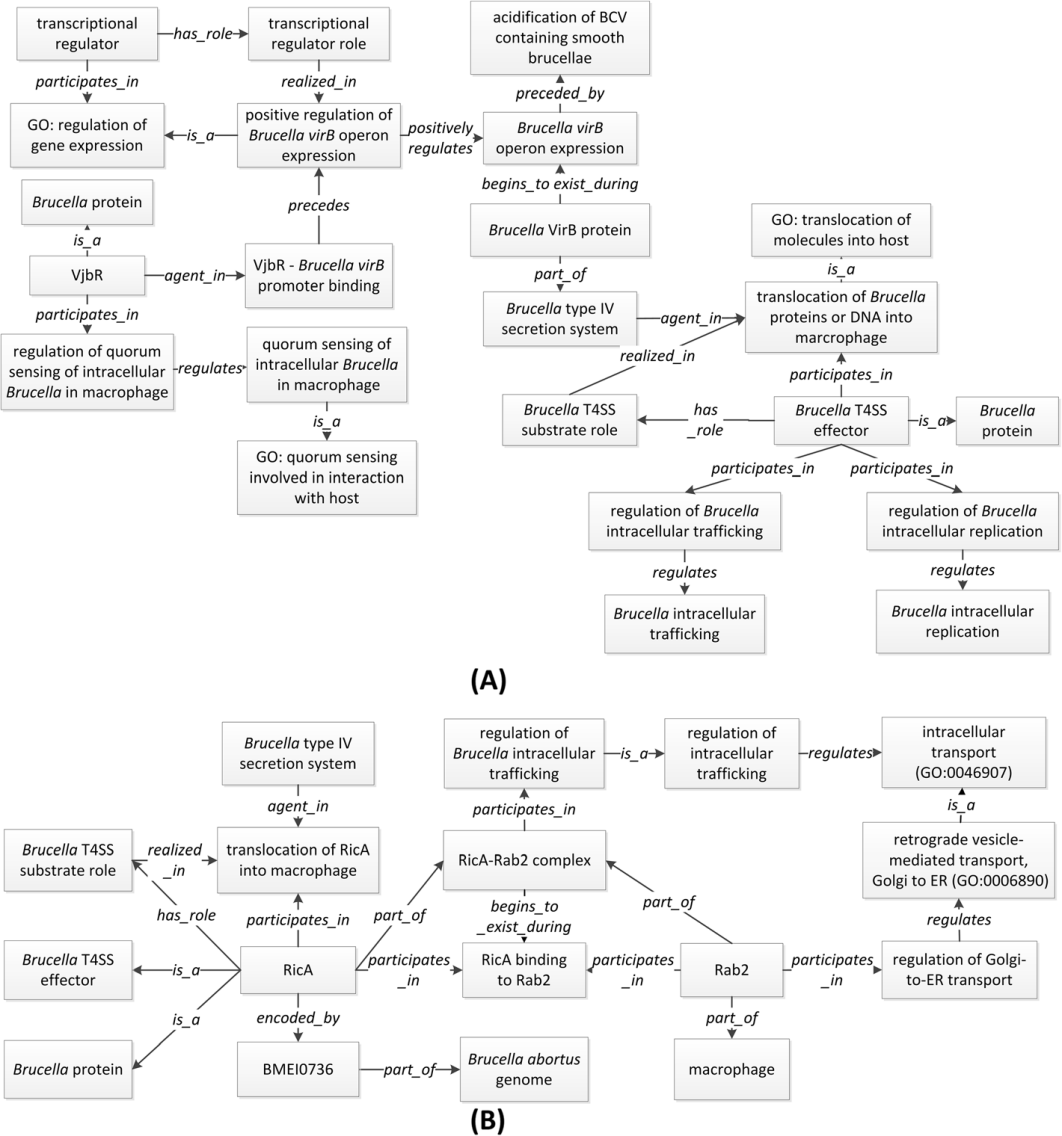
Ontological modeling of *Brucella* type IV secretion system and its effects. **a** The general *Brucella* type IV secretion in *Brucella* (**a**) and example of RicA as T4SS effector (**b**)

Figure 4a represents *Brucella* T4SS and its roles in the pathogenesis of smooth *Brucella*. The ‘*Brucella virB* operon expression’ is preceded by ‘acidification of smooth *Brucella* containing phagosome’. The *Brucella* VirB proteins, encoded by the *Brucella virB* operon, start to exist with the expression of the *virB* operon genes (begin_to_exist_during). The process of binding a *virB* promoter regulates the T4SS *virB* operon expressions. For example, VjbR, a LuxR-type quorum-sensing regulator, binds on the *virB* promoter, and activates *Brucella virB* operon expression [42]. Therefore, VjbR regulates *Brucella* T4SS directly and subsequently has an impact on the effectors of T4SS protein secretions. As shown in Fig. 4a, VjbR is an agent in the *Brucella virB* promoter binding process, which leads to positive regulation of the *Brucella virB* operon expression.

*Brucella* T4SS acts as a translocator of *Brucella* proteins or DNA into a macrophage [42]. Entities translocated or secreted by *Brucella* T4SS are termed as ‘*Brucella* T4SS effectors’, which regulate different processes essential to *Brucella* intracellular trafficking or replication. A *Brucella* T4SS effector is defined as “a molecular entity that bears the *Brucella* T4SS effector role” [40]. The ‘*Brucella* T4SS effector role’ is a new IDOBRU term that is defined as: “a role that inheres in a *Brucella* protein or a DNA upon which the *Brucella* T4SS acts, and as a result, the protein or DNA is secreted out of the bacterium.”

As an example of a *Brucella* T4SS effector, *Brucella* protein RicA (Rab2 interacting conserved protein A), is represented in IDOBRU to illustrate the *Brucella* T4SS virulence mechanism (Fig. 4). *Brucella* RicA, encoded by a *Brucella* gene BMEI0736, binds to human small GTPase protein Rab2 [43]. RicA is translocated from *B. abortus* to infected macrophages. However, this phenomenon does not occur when a *Brucella virB* mutant infects the macrophage cell. Rab2 also coordinates the retrograde Golgi-to-ER transport [44]. The Rab2 is essential for the formation of the fusion between BCV and ER, which results in the *Brucella* replication niche [45]. As shown in Fig. 4b, *Brucella* T4SS participates in the ‘process of translocation of RicA into a macrophage’ as an agent. *Brucella* protein RicA bears ‘*Brucella* T4SS effector role’, and it participates in the processes of ‘translocation of RicA into macrophage’ and ‘RicA-Rab2 binding’. The process of ‘RicA binding to Rab2’ leads to the formation of ‘RicA-Rab2 complex’. Rab2 participates in the regulation of Golgi-to-ER transportation, which regulates the ‘retrograde Golgi-to-ER transport’. Overall, Fig. 4b provides a detailed explanation to an axiom stated in Fig. 4a: ‘*Brucella* T4SS effector participates in the regulation of *Brucella* intracellular trafficking’.

#### Representing Brucella erythritol metabolism

As described earlier, one of the intracellular environmental stresses that *Brucella* faces is nutritional deprivation. *Brucella* uses alternative metabolism pathways to obtain carbon, nitrogen, oxygen, phosphorus, sulfur and metals from its intracellular host [5]. For example, an alternative pathway for *Brucella* to acquire carbon is through the erythritol metabolism [28, 46]. The genome of attenuated *B. abortus* vaccine strain S19 includes a 703 nucleotide deletion on its *ery* operon. The deletion affects the gene *eryC* coding for an enzyme erythrulose-1-phosphate dehydrogenase (EryC) and another gene *eryD* that encodes for EryD, a regulator of *ery* operon expression [47]. The deletion is a cause of the attenuation characteristic of strain 19 [48]. The *eryC* mutant of *B. suis* also reduces its intracellular replication in macrophage cells [46].

**Fig. 5:**
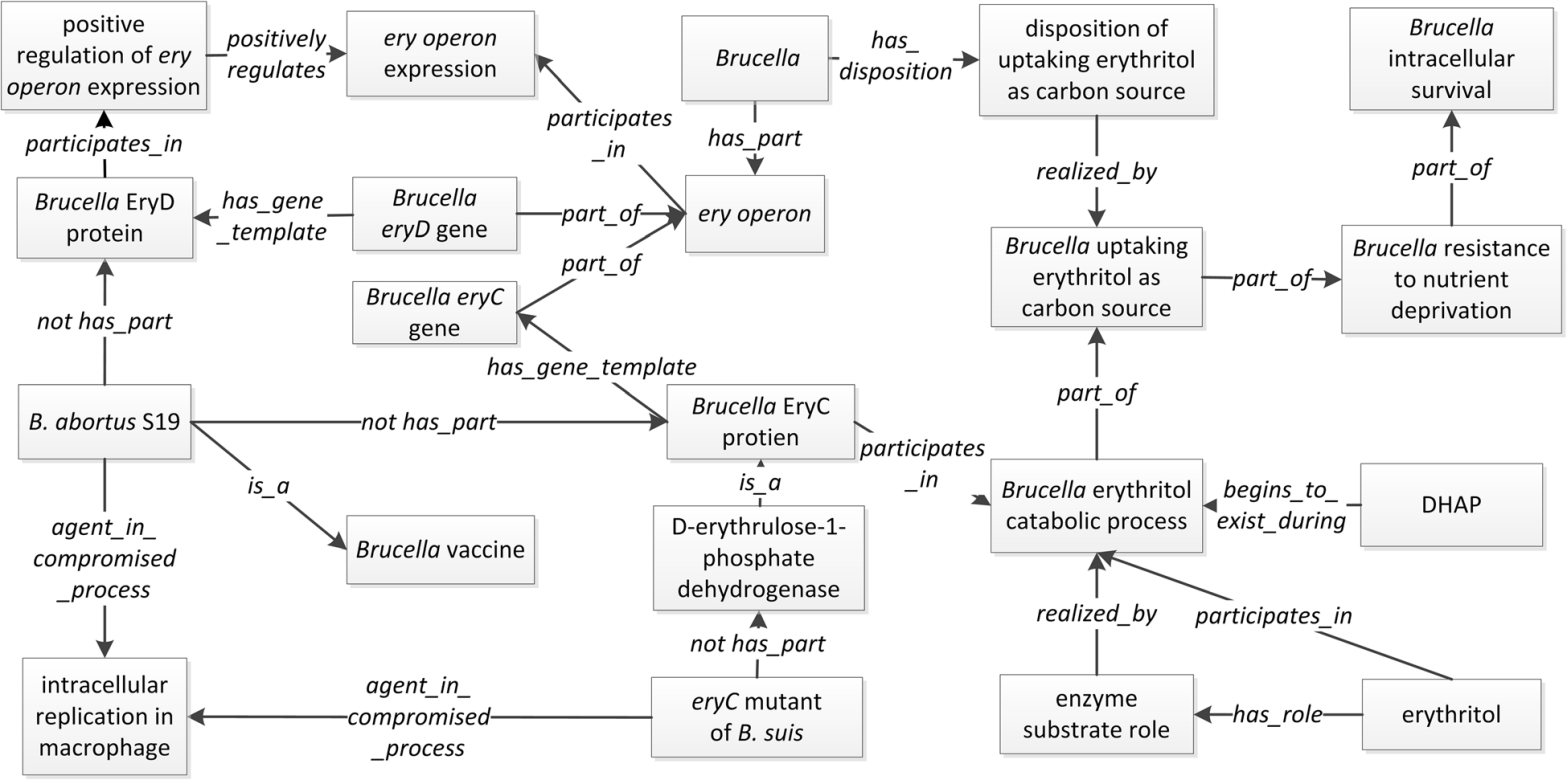
Ontological representation of *Brucella* erythiritol metabolism and its involvement in *Brucella* pathogenesis

To ontologically represent the virulent characteristic of EryC, we started by representing the whole process and its participants at the molecular level and organism level (Fig. 5):

1. The erythritol metabolism pathway is important for *Brucella* intracellular replication. The intracellular *Brucella* in a macrophage has the disposition of uptaking erythritol as the carbon source, which is realized in the relevant ‘uptaking erythritol process’. The process of ‘uptaking erythritol as carbon source’ has ‘*Brucella* erythritol catabolic process’ as its part. The ‘enzyme substrate role’ of erythritol is realized in the erythritol catabolic process. The term ‘enzyme substrate role’ is defined as “A role that inheres in a protein or a compound upon which enzyme catalyzes. It is realized in the enzymatic reaction processes, where the molecules at the beginning of the process, called substrates, are converted into different molecules, namely products.” The final product of ‘erythritol catabolic process’ is the dihydroxyacetone phosphate (DHAP), which is represented using the relation **begins_to_exist_during** as: ‘DHAP begins_to_exist_during erythritol catabolic process’. The process of ‘uptaking erythritol as carbon source’ is a partial process of ‘*Brucella* resistance to nutrient deprivation’, which accordingly precedes and is required for the process of ‘*Brucella* intracellular replication’. The relation **preceded_by** is used to denote the relation between these two processes. Both ‘*Brucella* response to nutrient deprivation’ and ‘*Brucella* intracellular replication’ are parts of the ‘host-*Brucella* interaction’ process (Fig. 5). It is noted that we did not model the detailed chemical reactions that are components of the whole erythritol catabolic process, because it is out of the scope of current IDOBRU development.
2. *Brucella* genes *eryC* and *eryD* involve in the erythritol metabolism pathway.
  Both *eryC* and *eryD* are parts of the *Brucella ery operon*. The *eryC* gene encodes erythrulose-1-phosphate-dehydrogenase enzyme (EryC), and it participates in the erythritol catabolic process. The *eryD* gene encodes for an EryD protein that regulates the *ery operon* expression [47]. The object property **regulates** is a relation between processes, and it is used in GO to represent the fact that a process *A* has a direct influence on another process *B* such that it controls some aspects of how process B unfolds [49]. Therefore, the protein EryD **regulates** the *ery operon* expression (Fig. 5).
3. *B. abortus* vaccine strain S19 has a 703-nucleotide deletion which interrupts both the coding regions of *eryC* (BAB2_0370) and *eryD* (BAB2_0369) [48]. The deletion affects the C terminal part of the *Brucella* protein EryC and the N-terminal part of the *Brucella* protein EryD [48]. Therefore, S19 lacks the intact EryD protein and EryC protein as its parts. In the other example, the *eryC* mutant of *B. suis* has no EryC protein as its part, and it is an agent in the process of ‘reduced intracellular replication in macrophage’ [46]. ‘Reduced intracellular replication in macrophage’ is a compromised intracellular replication process in macrophage.

### Representing host immune responses to *Brucella* infection

Virulent *Brucella* is a stealthy bacterium that hijacks many host immune mechanisms to serve its own survival and replication inside a host [6]. As introduced above in the *Brucella* pathogenesis section, virulent *Brucella* is able to replicate inside macrophages which are typically powerful innate immune cells. *Brucella* can survive in replicative phagosomes inside macrophages where nutrients are difficult to obtain. The *Brucella*-containing phagosome does not fuse with bactericidal lysosomes [6]. Furthermore, to maintain the bacterial natural living niche, virulent *Brucella* prevents the programmed macrophage cell death. However, live attenuated *Brucella* strains, including *Brucella* cattle vaccine RB51 [50], induce apoptosis or other types of programmed cell death of infected macrophages, which destroys the *Brucella* living niche and exposes the bacteria to the most hostile extracellular environment [25, 34]. Therefore, the programmed cell death process benefits the host. Below we ontologically represent and analyze the process using the example of live attenuated rough *B. abortus* strain RB51, which is a cattle brucellosis vaccine licensed and used in the USA and many other countries [50].

**Fig. 6:**
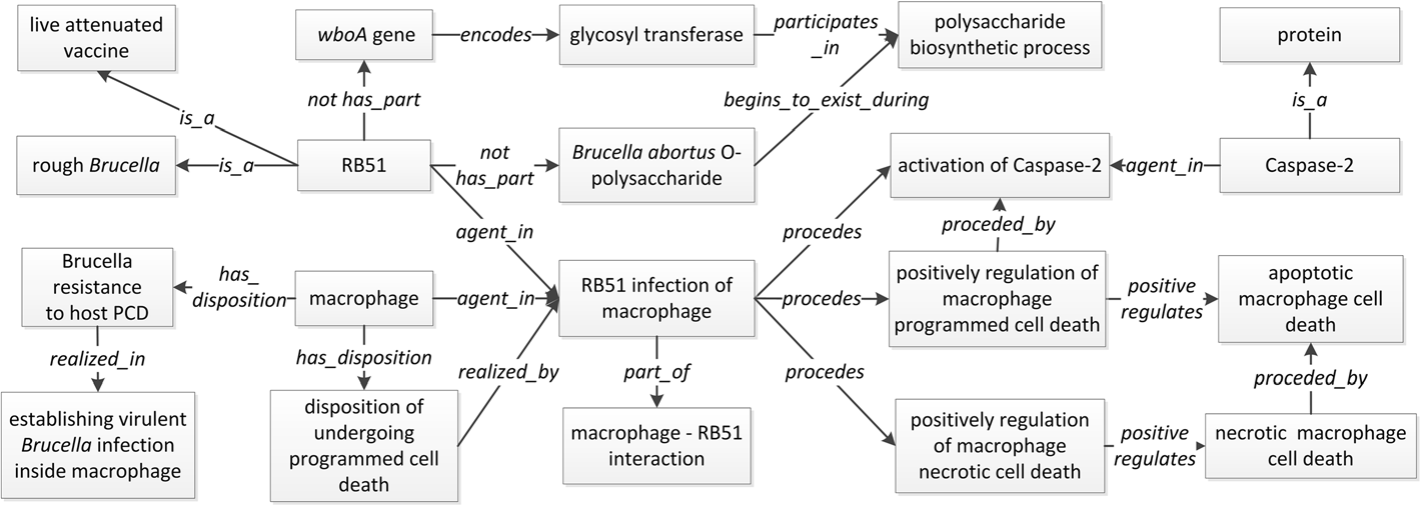
Ontological representation of *Brucella* vaccine strain RB51-induced macrophage cell death

Our previous wet-lab studies have shown that RB51 and many other rough attenuated *Brucella* strains induce caspase-2-mediated pro-inflammatory cell death in macrophages through the release of cytochrome *c* from mitochondria [34, 35]. RB51 has an insertion within *wboA* gene that leads to the deficiency of *Brucella* LPS O polysaccharide and results in its rough phenotype [51]. Figure 6 illustrates ontological representation of RB51-induced capspase-2-mediated macrophage cell death. In this representation, RB51 has no intact *wboA* gene that encodes for an enzyme involved in the biosynthesis of *Brucella abortus* O-polysaccharide [52]; therefore, RB51 is lack of *Brucella abortus* O-polysaccharide. RB51 is an agent in the process of its infecting a macrophage, which is a part of the ‘macrophage-RB51 interaction’ process. The ‘RB51 infection of macrophage’ triggers (*RO:precedes*) three processes: ‘activation of caspases-2’, ‘positive regulation of macrophage programmed cell death’, and ‘positive regulation of macrophage necrotic cell death’. The ‘activation of caspases-2’ leads to (*RO:precedes*) ‘positive regulation of macrophage programmed cell death’. The ‘positive regulation of macrophage cell programmed death’ positively regulates the ‘apoptotic macrophage cell programmed death’, which leads to ‘necrotic macrophage cell death’. The ‘necrotic macrophage cell death’ is positively regulated by the ‘positively regulation of macrophage necrotic cell death’.

From a macrophage’s perspective, the macrophage has two opposite dispositions: 1) the ‘disposition of undergoing programmed cell death’ that is realized by attenuated RB51 infection of macrophage; and 2) the ‘resistance to programmed cell death’ that is realized by ‘virulent *Brucella* infection of macrophage’.

### Ontological representation and queries of virulence factors and associated host-*Brucella* interactions

#### Ontology classification of Brucella host-Brucella interactions involving virulence factors

We previously defined a ‘*Brucella* virulence factor’ as “*virulence factor that bears Brucella virulence factor disposition*” [10]. The ‘*Brucella* virulence factor disposition’ is defined as “a disposition borne by a biological macromolecule produced by *Brucella* spp. that is the disposition to improve survival of the pathogen in a host, improve transmission of the pathogen to a host, or cause pathological processes in a host”. To further expand the definition, virulence factors are ontologically classified in this article to be critical to five pathogen virulence (or microbial pathogenesis) processes:

1. colonization of a niche in the host (this includes adhesion to cells);
2. evasion of the host’s immune response;
3. inhibition of the host’s immune response;
4. entry into and exit out of cells (if the pathogen is an intracellular one);
5. absorption of nutrition from the host.

As shown in the modeling of T4SS and *eryC* described above, a *Brucella* virulence factor is involved in at least one critical process as part of the host-*Brucella* interaction, or a process precedes the critical process. Although many *Brucella* molecules participate in the host-*Brucella* interaction processes, not all of them contribute to the virulence of the *Brucella*. One method to confirm the status of a molecule being a virulence factor is knock-out experimental evidence, where the pathogen without this molecule realizes a “reduced” or “abolished” virulence disposition during the host-*Brucella* interaction.

#### Ontology representations of Brucella virulence gene mutants

The major category of virulence factors are protein virulence factors that are encoded by virulence genes. Compared to the original IDOBRU that includes 245 *Brucella* virulence factors [10], current IDOBRU has been expanded to include 432 experimentally-verified *Brucella* virulence factors. All these virulence factors are experimentally verified. The gene mutation followed by experimental examination of the virulence of the gene mutant inside a host (i.e., the host organism or host cell) is the major method to detect the status of a pathogen protein as a virulence factor.

**Fig. 7:**
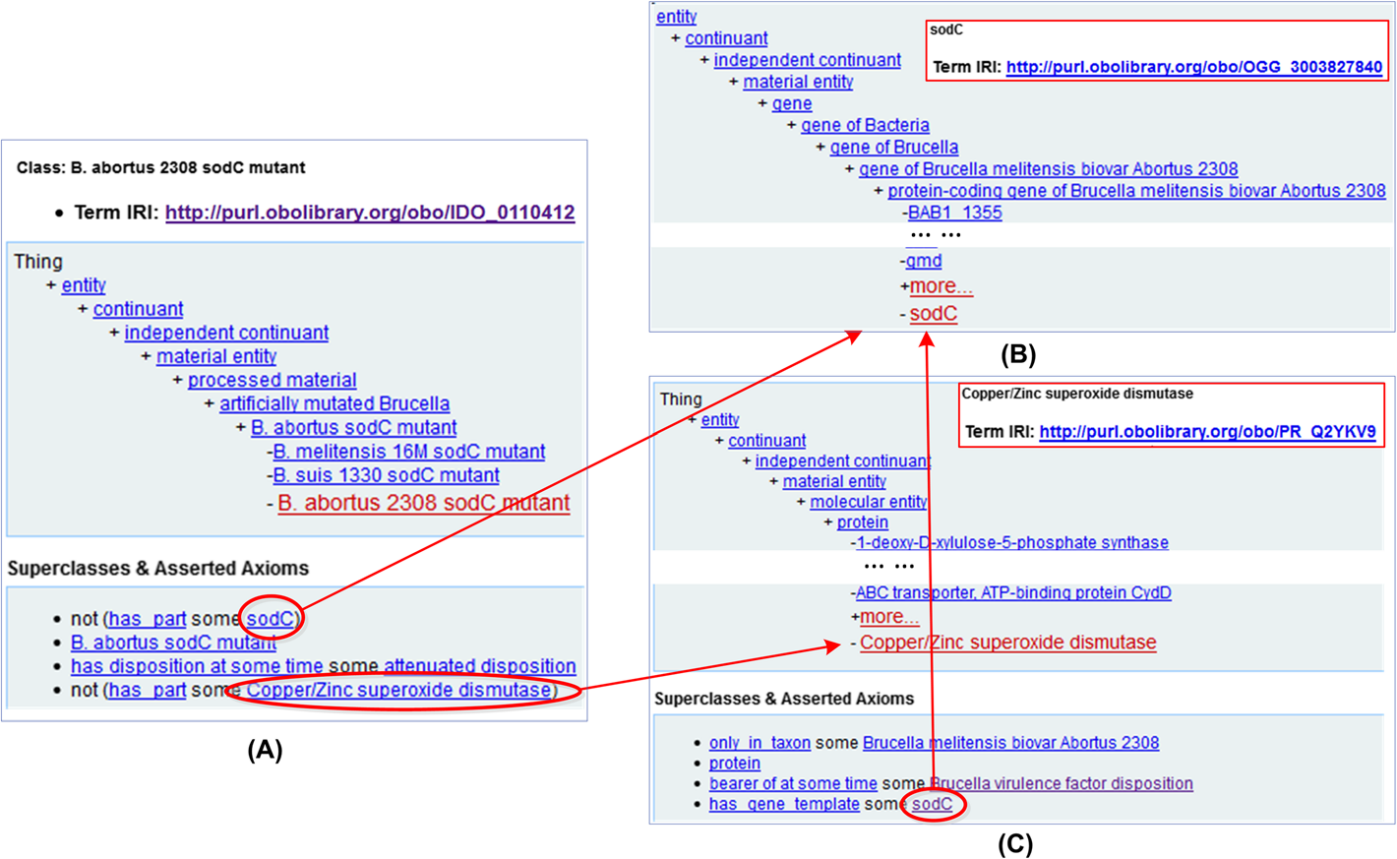
IDOBRU representation of a *Brucella* virulence gene mutant. The example shown here is *B. abortus* strain 2308 *sodC* mutant (**a**). This mutant has a mutation of strain 2038 *sodC* gene (**b**), which encodes a protein called Copper/Zinc Superoxide Dismutase (SOD) (**c**). The relation ‘has_gene_template’ is used to link the protein to the gene. The screenshots came from the IDOBRU page in Ontobee [61]

Figure 7 shows how IDOBRU represents a virulence gene mutant that lacks an intact protein virulence factor. Specifically, a gene mutant is represented in IDOBRU as a mutant that does **not has_part** a gene, which also results in the lack of an intact protein (Fig. 7a). The original IDOBRU used IDO ontology identifiers with the “IDO_” to represent *Brucella* genes and proteins. However, *Brucella* genes and proteins may be used in other ontologies such as the Vaccine Ontology (VO) [53, 54]. The usage of IDO-specific identifiers does not support data integration and resource interoperability. As detailed in the Methods section and shown in Fig. 7, in the new version of IDOBRU, we have imported the Ontology of Genes and Genomes (OGG) IDs (Fig. 7b) and Protein Ontology (PRO) IDs (Fig. 7c) to represent the genes and proteins of *Brucella* virulence factors. OGG is a relatively new ontology that represents specific genes in different species [19]. PRO is an ontology of protein entities [20]. To link the gene and protein entities, we have adopted the PRO relation ‘has_gene_template’ to represent a protein encoded by a gene (Fig. 7c), and the relation ‘encodes’ to represent a gene encoding a protein.

#### Description rules to define virulence factors

To establish logical reasoning for a virulence factor, we developed five description rules as defined below. In the formulation of these rules, *o* denotes an organism O, *g* and *g’* denote genetic materials, *e* denotes a molecular entity, *p* denotes a process, *i* denotes a host-pathogen interaction process, and *mo* denotes a mutant of O.

**(IR3)** IF *o***has_part***g,* ∩ *g***encodes***e,* THEN *o***has_part***e*

IR3 means that if an organism has part of a gene that encodes for a molecular entity (i.e., gene product such as protein), then this organism has part of the molecular entity.

**(IR4)** IF *mo***has_part***g’,* ∩ *g’***derives_from***g,* ∩ (*g’***not has_part***part of g)* ∩ *genome of o***has_part***g,* THEN *genome of mo***not has_part***g*

IR4 means that if a mutant of an organism has an artificially altered gene *g’* that is derived from *g*, either by an insertion or partial deletion, then the genome of the mutant has no intact *g* as its part. When the *g* is fully deleted (i.e., *g* gene knock-out) from mutant *mo*, the above rule will not apply. In this case, we simply assert that *genome of mo***not has_part***g* (see below).

**(IR5)** IF *genome of mo***not has_part***g,* THEN *mo***not has_part***g*

If the genome of mutant has no intact gene *g* as its part, the mutant has no *g* as its part.

**(IR6)** IF *genome of mo***not has_part***g,* ∩ *g***encodes***e,* THEN *mo***not has_part***e*

IR6 means that if the mutant has no intact gene *g* as its part, and the gene *g* encodes the molecular entity *e* in the non-mutated organism, then the mutant has no intact *e* as its part.

IR7 was given as a final inference rule for inferring a virulence factor:

**(IR7)** IF (*mo***has disposition at some time***attenuated disposition* ∩ *attenuated disposition***realized in***i*) ∩ *mo***not has_part***e,* ∩ (*mo***agent_in_compromised_process***p* ∩ *p***is_a***pathogen virulence process*), THEN *e***is_a***virulence factor*.

For example, as shown in Fig. 5, (*B. abortus eryC mutant***not has_part** EryC*)* AND *(B. abortus eryC mutant***agent_in_compromised_process***Brucella intracellular replication in macrophage*) AND (*Brucella intracellular replication in macrophage***is_a***pathogen virulence process)* means that *EryC***is_a***Brucella virulence factor.* According to IR6, if a gene *g* encoding a protein *e* is mutated from a mutant, the mutant does not have the intact protein *e* any more. Since *eryC* is mutated from the *eryC* mutant, we can infer that the *eryC* mutant does not have EryC.

We can therefore identify the biological process important to the pathogen virulence during the host-*Brucella* interaction. Using the same Fig. 5 example, (*EryC***participate_in***Brucella* erythritol catabolic process) AND (*EryC mutant of B. abortus***agent_in_compromised_process***intracellular replication in macrophage*), which means that the *Brucella* erythritol catabolic process is crucial to the intracellular replication in macrophage.

With the support of the above representations defined by IR3-7, we have annotated 269 virulence factors associated with various macrophage-*Brucella* interactions. IR7 is included in IDOBRU (Additional file 1: Figure S1).

#### SPARQL queries of IDOBRU for virulence factors critical for host-Brucella interactions

In this study, several SPARQL scripts were generated to query the information related to host-*Brucella* interactions. The details are provided below:

First, a simple SPARQL script was generated to query the number of protein virulence factors collected in IDOBRU (Additional file 2). Each virulence factor protein is ‘**bearer of at some time**’ (BFO_0000053) some ‘*Brucella* virulence factor disposition’ (IDO_0100116). The query identified 432 protein virulence factors.

**Fig. 8:**
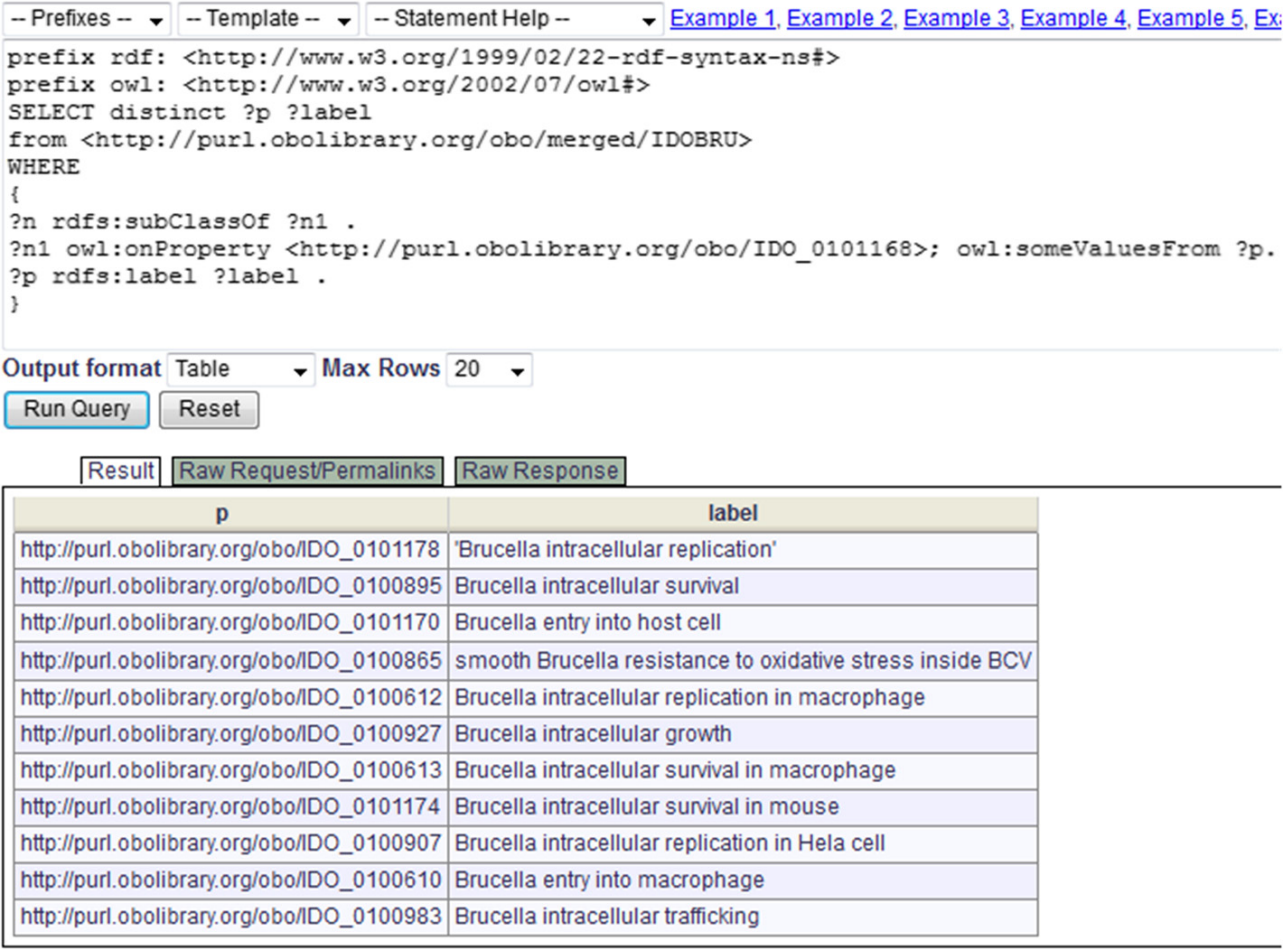
SPARQL query of biological processes involving *Brucella* virulence factors. This script queries the compromised *Brucella* processes in which *Brucella* virulence gene mutants participate. The query was performed using the Ontobee SPARQL web-interface (http://www.ontobee.org/sparql). The IDO_0101168 in the script is ‘agent_in_compromised_process’

The second SPARQL query identifies the processes in which *Brucella* virulence factors participate (Fig. 8). Specifically, the script queries what compromised processes *Brucella* virulence factor mutants get involved in. The relation ‘agent_in_compromised_process’ as described earlier is used here. In total, 11 biological processes, for example, ‘*Brucella* entry into macrophage’ (IDO_0100610) and ‘*Brucella* intracellular trafficking’ (IDO_0100983), were identified (Fig. 8). These processes are critical to *Brucella* pathogenesis inside host cells.

Third, those *Brucella* mutants that are attenuated inside macrophages during various macrophage-*Brucella* interactions were identified using SPARQL (Additional file 3). Each of these mutants is associated with a particular gene (represented in OGG) and a corresponding protein (represented in PRO) (Fig. 7). Therefore, the queries also provide us a way to extract those virulence genes and virulence factors important for macrophage-*Brucella* interactions. In total, 269 gene mutants that are associated with 269 genes and protein virulence factors were found. The list of all these gene mutants is also provided in Additional file 3.

Fourth, the *Brucella* protein virulence factors important for *Brucella* intracellular replication inside macrophages were detected using two SPARQL queries (Additional file 4). A query identified 81 such virulence factors, and the other query provided the detailed list of these factors (Additional file 4).

It is noted that the inference rules IR3-7 provide the logic clues on the second and third sets of queries. Specifically, to search *Brucella* virulence factors important for intracellular replication in macrophages, first we retrieved all possible gene mutants. Each protein encoded by a gene mutated in a mutant is an agent involved in the compromised process of ‘macrophage-*Brucella* interaction’ (IDO_0100832) (Additional file 3: Figure S3) or ‘*Brucella* intracellular replication in macrophage’ (IDO_0100612) (Additional file 4: Figure S4). The relation ‘agent_in_compromised_process’ as described earlier is used here. Due to the mutation event, the intact *Brucella* protein (virulence factor) does not exist in the mutant organism (IR4 and IR6). Therefore, we are able to retrieve all the corresponding virulence factor proteins using the **not has_part** relation (Additional file 4: Figure S4).

## Discussion

Compared to the original IDOBRU paper published in 2011 [10], current article included several novel contributions. First, while the original paper only includes one section with one figure in the topic of the host-*Brucella* interaction (i.e., virulence factor and pathogenesis), current article focuses on the modeling and analysis of different aspects of the host-*Brucella* interactions. Specifically, this article first ontologically differentiates smooth and rough *Brucella* phenotypes and how each phenotype is related to *Brucella* virulence. Six host-*Brucella* interaction subtypes are categorized, and the agents participating in any of the subtypes (i.e., macrophage-*Brucella* interaction) are defined. IDOBRU is also used to represent detailed processes of *Brucella* invasion, trafficking, and replication inside host cells. Examples described in this article include two major *Brucella* pathogenesis mechanisms: the Type IV secretion system (T4SS) and the erythritol metabolism. In terms of host immune response against *Brucella* infection, the inhibition or promotion of host programmed cell death is specifically modeled using IDOBRU. Regarding the virulence factors, this article for the first time ontologically classifies five different types of host-pathogen interaction processes where virulence factors may play a critical role. Approximately 200 more virulence factors have been included in current IDOBRU since the original IDROBRU publication. Second, seven specific inference rules are generated and described in this paper for reasoning related to host-*Brucella* interactions and *Brucella* virulence factors. The layout and implementation of these inference rules provide more power in using IDOBRU for computer-assisted reasoning. Third, this article introduces an updated style of representing *Brucella* genes and proteins. Instead of using IDO IDs to represent genes and proteins, the updated IDOBRU imports OGG and PRO IDs for more authentic representations of *Brucella* genes and proteins. Such gene/protein representations support ontology reuse and interoperability. Fourth, this article provides many SPARQL scripts that demonstrate the applications of IDOBRU. Furthermore, many more ontology terms have been added to IDOBRU. Compared to the original IDOBRU version published in 2011 that includes 1503 terms, current version 1.2.79 includes 3488 terms. We have more than doubled the numbers of the terms in the IDOBRU ontology, clearly showing our progress in the IDOBRU development.

Other literature reports exist for ontological modelling of host-pathogen interactions. The Plant-Associated Microbe Gene Ontology (PAMGO) Consortium uses GO for modeling host-pathogen interactions based on the investigation of plant-associated symbionts [55]. Their efforts yielded a group of GO biological process terms that capture the processes occurring between hosts and symbionts (from mutualists to pathogens). PAMGO is focused on representing processes. Representation of host participants (e.g., organelle like ER, and cell membrane) and pathogen details (e.g., LPS of *Brucella*, *Brucella* proteins) was not covered. IDO-core provides many top level terms in the area of host-pathogen interactions, such as establishment of localization in host (IDO_0000625). As an extension ontology of IDO-core, the Malaria Ontology team developed several terms related to malaria-host interact processes, such as ‘inhibition of invasion’ and ‘responsiveness to host cue’ [56]. Another ontology named Host Pathogen Interaction Ontology (HPIO) found on NCBO BioPortal is current under development, which aims to describe the host-pathogen interactions between *Salmonella* bacteria and swine, also as an extension of IDO-core. However, HPIO does not represent the interactions between host and pathogens extensively. None of the above efforts covers the participants of the interaction processes and relations between those processes. In contrast, IDOBRU represents various participants in the interaction processes between *Brucella* and its hosts with details at the organism, cell, and molecular levels. IDOBRU is used as a framework to model different proteins, complexes and interaction processes such as how virulence factors play a role in the *Brucella*-host interactions and how the interactions happened as a series of sequential events. To support data standardization and exchange, formal relations are applied in the IDOBRU modeling. To the best of our knowledge, our IDOBRU modeling and representation of various areas of host-*Brucella* interactions represent the first comprehensive host-pathogen interaction ontological analysis.

The host-*Brucella* interaction is modeled in IDOBRU as a process with many specific interaction subclasses. The interactions between *Brucella* and hosts are *Brucella-*specific and host (e.g., host organism or cell) - specific. For example, the pathway of *Brucella* entry into macrophage differs from the bacterial entry into epithelial cell, and the immune response and intracellular trafficking induced by *Brucella* infection differ among professional and non-professional phagocytes [57]. Therefore, it is important to classify different subtypes of host-*Brucella* interactions by host cells. The macrophages are emphasized in this study since macrophage is likely the most critical host cell type in terms of host-*Brucella* interactions [32]. More ontological representations with other host cells are needed. Similarly, we will need to classify the subtypes based on host organism or host organs as well, since the infection of any *Brucella* strain has its host preference. These aspects are critical to the development of host-specific *Brucella* vaccines.

Compared to well-studied model organisms such as *E. coli*, *Brucella* is less studied, and the coverage of *Brucella* research is often unbalanced and limited. For example, middle products and enzymes involved in the erythritol catabolism pathway in *Brucella* are not available in PRO or ChEBI. We communicated the PRO and ChEBI groups and submitted related middle product terms to ChEBI and enzyme terms to PR. For example, over 1000 *Brucella* strain specific proteins were submitted to PR. Although the enzyme databases and knowledge bases are well developed and contain authentic comprehensive information, the erythritol catabolic pathway is not included in well-known enzyme databases such as BRENDA (http://www.brenda-enzymes.org/). GO has very few gene products from *Brucella* annotated with experimental evidence codes (http://www.geneontology.org/GO.evidence.shtml).

The inference rules (IR3-IR7) generated in this study provide a framework on inferring virulence factors. These rules can be used for validating the ontology and populating the ontology. These inference rules can also be generalized to other pathogen. The inference rules represent knowledge and provide a format for computer-understandable automated reasoning. These rules offer explicit and transparent assumptions for representing virulence factors in the case of the host-*Brucella* interaction. Based on the inference rules and available data, a computer will be able to assess if a material entity is a specific virulence factor. Since the mechanisms of virulence are different from species to species, more inference rules may be developed with different types of pathogens (e.g., HIV virus).

SPARQL provides a powerful method for querying and analyzing the data in an ontology [58]. For example, our queries identified 269 protein virulence factors related to macrophage-*Brucella* interactions; and among these proteins, 81 are important for intracellular replication within macrophage from the knowledge stored in current IDOBRU. Note that *virB1*, *virB5*, *virB8*, *virB9* and *virB10* are in the above list, which validates the modeling of T4SS mechanism in this paper. In addition, we have identified 11 biological processes important for *Brucella* virulence (Fig. 8). These simple but powerful SPARQL queries demonstrate the applications of IDOBRU and IDOBRU-based SPARQL technology.

One important contribution of this paper is its first report in co-representing genes and proteins using the Ontology of Genes and Genomes (OGG) [19] and the Protein Ontology (PRO) [20]. The majority of *Brucella* virulence factors are proteins, which are encoded by specific genes in different *Brucella* strains. The practice of using IDOBRU IDs in our original IDOBRU version was not ideal since it does not support ontology reuse and integration. To address this issue, the new version of IDOBRU uses the gene and protein IDs from the OGG and PRO, two ontologies in the OBO ontology library. The OGG ontology was recently developed to represent genes from specific organisms by reusing existing resources, primarily the NCBI Gene resource [59]. Due to the large numbers of genes available in different organisms, OGG includes different subsets, each of which represents genes from one or a few organisms. Using the OGG development strategy, we first generated the OGG *Brucella* subset that covers all genes of three major *Brucella* strains. All virulence factor genes covered in IDOBRU are from these three *Brucella* strains. The availability of the OGG *Brucella* subset allows us to retrieve and reuse the OGG terms to represent *Brucella* genes in IDOBRU. Similarly, specific *Brucella* proteins were generated in PRO and reused in IDOBRU. Furthermore, using two object properties (i.e., ‘has_gene_template’ and ‘encodes’), we were able to represent the relations between genes and proteins. Since genes and proteins are two fundamental entities in biology, this study provide a demonstration on how these can be ontologically represented and interlinked.

## Conclusions

In this paper, we ontologically represent various *Brucella-*host interactions primarily using the *Brucella*-macrophage interaction as a use case. A formal definition of the *Brucella*-host interaction was given in OWL format. After the definitions on smooth and rough *Brucella* are given, six subtypes of *Brucella*-host interactions are classified according to the *Brucella* phenotypes and host cell types. IDOBRU is further used as a platform to represent interactive processes including *Brucella* invasion, intracellular trafficking and intracellular replication at an organism level. By representing the *Brucella* pathogenesis mechanisms using *Brucella* T4SS and EryC examples, we demonstrate how to ontologically link biological processes from the organism level down to the molecular level. Description logical inference rules have also been defined to infer: 1) the interaction process between two species (i.e., a host and a pathogen); 2) the temporal relations of biological processes; 3) relations between gene, protein, genome, and gene mutant; and 4) a virulence factor. For this study, many new terms have been added into IDOBRU. Using SPARQL queries generated based on inference rules, out of the 269 virulence factors related to macrophage-*Brucella* interactions, 81 virulence factors were found to be important for *Brucella* intracellular replication inside macrophage. Eleven biological processes were also found important for *Brucella* virulence.

## Methods

### Ontology editing

The format of W3C standard Web Ontology Language (OWL2) (http://www.w3.org/TR/owl-guide/) was applied for IDOBRU development. The Protégé OWL ontology editor (http://protege.stanford.edu/) (versions 4.3 and 5.0 beta) was used to edit IDOBRU.

### Existing ontology term import

The ontology development uses a hybrid bottom-up and top-down method as described in our original IDOBRU article [10]. For this host-*Brucella* interaction study, many external ontology terms from existing ontologies, including Cell Type Ontology (CL) [14], Chemical Entities of Biological Interest (ChEBI) [15], Gene Ontology (GO) [16], Protein Ontology (PRO) [20], were imported to IDOBRU using OntoFox (http://ontofox.hegroup.org/) [31].

### IDOBRU access and visualization

The latest version of IDOBRU is always available at Sourceforge website: (http://svn.code.sf.net/p/idobru/code/trunk/src/ontology/brucellosis.owl). Not that this is an unmerged OWL file, and it imports many other OWL files in the same folder. Therefore, it would be best to get all the related ontology files via SVN. IDOBRU has also been deposited in the NCBO BioPortal (http://bioportal.bioontology.org/ontologies/IDOBRU) and the Ontobee linked ontology browser system (http://www.ontobee.org/browser/index.php?o=IDOBRU). Both NCBO BioPortal and Ontobee provide interactive search and visualization features for IDOBRU exploration and analysis.

### OGG and PRO representation of *Brucella* virulence factors

The *Brucella* subset of the Ontology of Genes and Genomes (OGG) was generated using a method described in the OGG paper [19]. Specifically, a NCBITaxon subset was generated to include three *Brucella* strains using OntoFox [31]. These strains are *B. abortus* strain 2308, *B. suis* strain 1330, and *B. melitensis* strain 16 M. All annotated *Brucella* genes in IDOBRU come from these three strains. Most of the information of all added *Brucella* genes encoding protein virulence factors was obtained from the manually annotated Victors database (http://www.phidias.us/victors) in the PHDIAS resource [60]. The OGG *Brucella* subset was submitted to the He group RDF triple store [61]. OntoFox was then used to retrieve the *Brucella* genes covered in IDOBRU.

The corresponding proteins encoded by these *Brucella* genes are represented by Protein Ontology (PRO) [20]. OntoFox was used to extract the information of these proteins from PRO. The resulting PRO subset was then imported to IDOBRU.

### Queries of IDOBRU

SPARQL scripts were developed to query IDOBRU using the IDOBRU SPARQL query web page (http://www.phidias.us/bbp/idobru/sparql/index.php) located in the *Brucella* Bioinformatics Portal (BBP; http://www.phidias.us/bbp) [60, 62].

### Implementation of inference rules

The reasoner HermiT 1.3.8 (http://hermit-reasoner.com/) as a plugin in the Protégé OWL editor (http://protege.stanford.edu/) was used to implement the inference rules defined in this paper. The rule view editor in the Protégé OWL editor was used to edit the rules. The ontology rule view in Protégé is accessible from the Protégé menu Window → Views → Ontology views → Rules. The saved IDOBRU OWL file contains the rules in the format of OWL with SWRL codes.

## Additional files

Additional file 1:
**Implementation of inference rules using the Protégé platform.** (PDF 383 kb) Additional file 2:
**SPARQL query of IDOBRU for the total number of protein virulence factors in IDOBRU.** (PDF 309 kb) Additional file 3:
**SPARQL query of IDOBRU for** ***Brucella*** **mutants that are attenuated inside macrophages during the macrophage-** ***Brucella*** **interactions.** Since each mutant is associated with one gene and one protein, these queries also allow us extract those virulence genes and protein virulence factors that participate in various macrophage-*Brucella* interactions. (PDF 353 kb) Additional file 4:
**SPARQL query of IDOBRU for** ***Brucella*** **protein virulence factors important for the intracellular replication of** ***Brucella*** **inside macrophages.** (PDF 198 kb)
