# Supplementary material for: Ontology-based representation and analysis of host-Brucella interactions
Source: J Biomed Semantics. 2015 Oct 5;6:37. doi: 10.1186/s13326-015-0036-y (PMC4594885; doi:10.1186/s13326-015-0036-y)
Supplement: Additional file 1: — Implementation of inference rules using the Protégé platform. (PDF 383 kb) [file 13326_2015_36_MOESM1_ESM.pdf]

**Supplemental file – Rule Testing**

**Note:** This supplemental document provides the information of how we evaluated the inference rules generated in the manuscript. The description about these inference rules can be found in the manuscript text.

These inference rules were tested using Protégé OWL editor version 4.3 or 5.0. The HermiT (version 1.3.8) reasoner on the Protégé editor was used to test these inference rules.

**Inference Rule 1 (IR1):**

If (a agent_in p, ∩ b agent_in p), ∩ p is_a biological_process, ∩ (a part_of A, ∩ b part_of B), ∩ (A is_a (host organism ∪ host cell), ∩ B is_a Brucella), then p is_a 'host-Brucella interaction'

<u>Rule implemented in Protégé OWL editor (a simplified version):</u>

biological_process(?p),
agent_in(?a,?p),
agent_in(?b,?p),
part_of(?a,?A),
part_of(?b,?B),
Brucella(?B),
'Brucella host'(?A)
-> 'host-Brucella interaction'(?p)

**Inference Rule 2 (IR2):**

If c initially participates in p2, ∩ c begins_to_exist_during p1, then p2 starts_during p1

<u>Rule implemented in Protégé OWL editor:</u>

'molecular entity'(?c),
biological_process(?p1),
biological_process(?p2),
'initially participates in'(?c, ?p2),
'begins to exist during'(?c, ?p1)
-> starts_during(?p2, ?p1)

**Inference Rule 7 (IR7):**
Note: IR7 incorporates the IR3-IR6 rules.

IF (*mo* ‘has disposition at some time’ ‘attenuated disposition’ ∩ ‘attenuated disposition’ ‘realized in’ *i*) ∩ *mo* (not ‘has part’) *e*, ∩ (*mo* agent_in_compromised_process *p* ∩ *p* is_a ‘pathogen virulence process’), THEN *e* is_a ‘virulence factor’

<u>Rule implemented in Protégé OWL editor:</u>

agent_in_compromised_process(?mo,?p),
'Brucella virulence process'(?p),
host-Brucella interaction(?i),
(not ('part of Brucella mutant'))(?e),
'has disposition at some time'(?mo,'attenuated disposition')
-> 'virulence factor'(?e)

**Protégé Screenshot:**

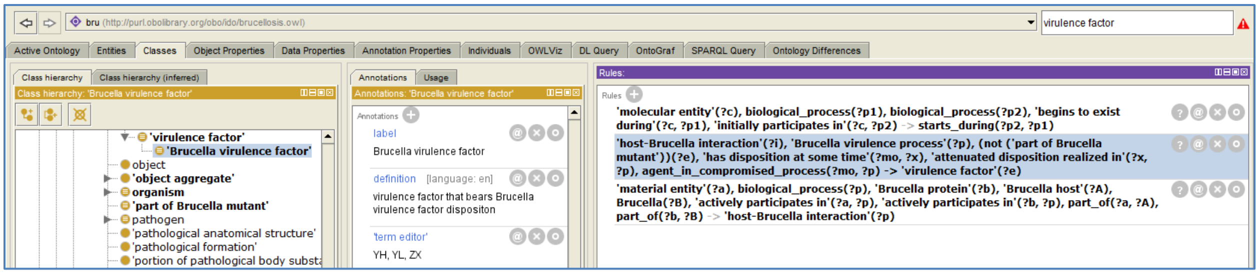


**Note:** To include the Rules view on Protégé, first open IDOBRU in Protégé, then on the Protégé menu, click using the following order: Windows → Views → Ontology views → Rules.

**Consistent tests:**

On 03/24/2015:

- Running environment: Proege 5.0, HermiT 1.3.8 classified in 2583685ms,
- Testing result: The ontology was consistent
