# Supplementary material for: Ontology-based representation and analysis of host-Brucella interactions
Source: J Biomed Semantics. 2015 Oct 5;6:37. doi: 10.1186/s13326-015-0036-y (PMC4594885; doi:10.1186/s13326-015-0036-y)

Supplemental file 2 – SPARQL query of the Number of *Brucella* Protein Virulence Factors

The following SPARQL script queries how many *Brucella* protein virulence factors are represented in IDOBRU.

SPARQL script:

```
prefix rdf: <http://www.w3.org/1999/02/22-rdf-syntax-ns#>
prefix owl: <http://www.w3.org/2002/07/owl#>
PREFIX obo: <http://purl.obolibrary.org/obo/>
SELECT (count(distinct ?n) as ?count)
from <http://purl.obolibrary.org/obo/merged/IDOBRU>
WHERE
{
?n rdfs:subClassOf ?n1  .
?n rdfs:subClassOf obo:PR_000000001 .
?n1 owl:onProperty obo:BFO_0000053 ; owl:someValuesFrom obo:IDO_0100116 .
}
```

Explanation of ontology terms in the SPARQL script:

obo:PR_000000001 – PR term 'protein'
obo:BFO_0000053 – BFO term 'bearer of at some time'
obo:IDO_0100116 – IDO term 'Brucella virulence factor disposition'.

Screenshot:

The following screenshot was generated using the Ontobee SPARQL program: http://www.ontobee.org/sparql

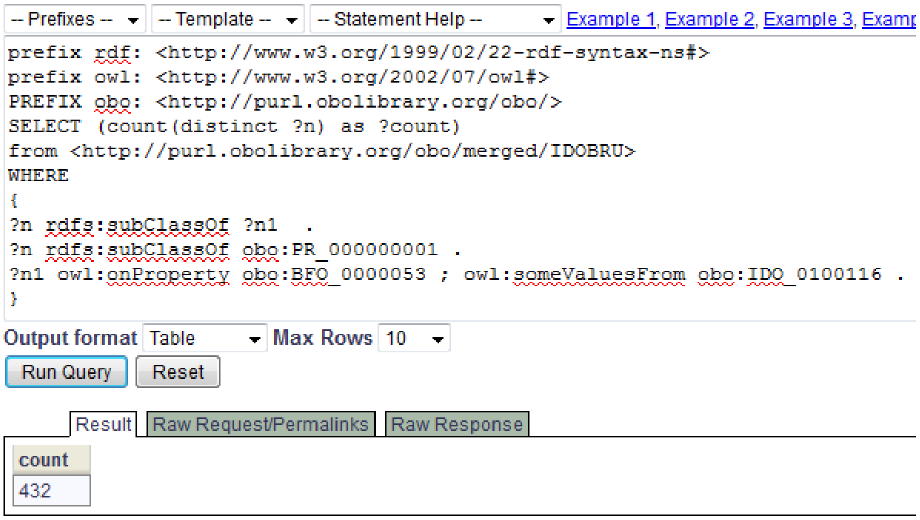

Supplement: Additional file 2: — SPARQL query of IDOBRU for the total number of protein virulence factors in IDOBRU. (PDF 309 kb) [file 13326_2015_36_MOESM2_ESM.pdf]
