# Supplementary material for: Ontology-based representation and analysis of host-Brucella interactions
Source: J Biomed Semantics. 2015 Oct 5;6:37. doi: 10.1186/s13326-015-0036-y (PMC4594885; doi:10.1186/s13326-015-0036-y)
Supplement: Additional file 3: — SPARQL query of IDOBRU for Brucella mutants that are attenuated inside macrophages during the macrophage- Brucella interactions. Since each mutant is associated with one gene and one protein, these queries also allow us extract those virulence genes and protein virulence factors that participate in various macrophage-Brucella interactions. (PDF 353 kb) [file 13326_2015_36_MOESM3_ESM.pdf]

Supplemental file 3 – SPARQL queries of *Brucella* mutants that are attenuated inside macrophages during the macrophage-*Brucella* interactions

This supplemental file includes two scripts, both related to the queries of *Brucella* mutants that are attenuated inside macrophages during the macrophage-*Brucella* interactions.

- Query 1: SPARQL query of **the total number of** *Brucella* mutants that are attenuated inside macrophages during a macrophage-*Brucella* interaction (IDO_0100832).
- Query 2: SPARQL query of **the list of** *Brucella* mutants that are attenuated inside macrophages during a macrophage-*Brucella* interaction (IDO_0100832).

Note: each mutant is associated with one gene and one protein. Therefore, these queries also found those virulence genes and protein virulence factors that participate in various macrophage-*Brucella* interactions.

```
##############################
#this query was running on http://sparql.hegroup.org/sparql
# Date: on 3/23/2015
# IDOBRU version: 86
#
##############################

#SPARQL query 1: the total number of Brucella mutants that are attenuated inside macrophages during a macrophage-Brucella interaction
#
# IDO_0100879 attenuated disposition;
# IDO_0100832 macrophage-Brucella interaction;
# BFO_0000051 has part
# BFO_0000112 has disposition at some time
# BFO_0000054 realized in

prefix rdf: <http://www.w3.org/1999/02/22-rdf-syntax-ns#>
prefix owl: <http://www.w3.org/2002/07/owl#>

SELECT (count(distinct ?s) as ?count)
from <http://purl.obolibrary.org/obo/merged/IDOBRU>

WHERE {
?s rdfs:subClassOf ?n6 .
?n6  owl:complementOf  ?n7.
?n7 owl:onProperty <http://purl.obolibrary.org/obo/BFO_0000051>; owl:someValuesFrom ?p.
{
 SELECT ?s
 WHERE
 {
 ?n rdfs:subClassOf ?n1 .
 ?n1 owl:onProperty <http://purl.obolibrary.org/obo/BFO_0000112>; owl:someValuesFrom ?n2.
 ?n2 owl:intersectionOf ?n3 .
```

```
 ?n3 rdf:first <http://purl.obolibrary.org/obo/IDO_0100879>; rdf:rest ?n4 .
 ?n4 rdf:first ?n5 .
 ?n5 owl:onProperty <http://purl.obolibrary.org/obo/BFO_0000054>; owl:someValuesFrom
<http://purl.obolibrary.org/obo/IDO_0100832> .
 ?s rdfs:subClassOf ?n .
 }
}
FILTER REGEX(str(?p), "PR") .
}

##############################
#Result: (as returned by the server on 3/24/2015 12:18pm)
count
269
##############################

#SPARQL query 2: the list of Brucella mutants that are attenuated inside macrophages during a
macrophage-Brucella interaction
#
# IDO_0100879 attenuated disposition;
# IDO_0100832 macrophage-Brucella interaction;
# BFO_0000051 has part
# BFO_0000112 has disposition at some time
# BFO_0000054 realized in

prefix rdf: <http://www.w3.org/1999/02/22-rdf-syntax-ns#>
prefix owl: <http://www.w3.org/2002/07/owl#>

SELECT distinct ?s ?l

from <http://purl.obolibrary.org/obo/merged/IDOBRU>

WHERE {
?s rdfs:subClassOf ?n6 .
?n6  owl:complementOf  ?n7.
?n7 owl:onProperty <http://purl.obolibrary.org/obo/BFO_0000051>; owl:someValuesFrom ?p.
?s rdfs:label ?l .
{
 SELECT ?s
 WHERE
 {
 ?n rdfs:subClassOf ?n1 .
 ?n1 owl:onProperty <http://purl.obolibrary.org/obo/BFO_0000112>; owl:someValuesFrom ?n2.
 ?n2 owl:intersectionOf ?n3 .
 ?n3 rdf:first <http://purl.obolibrary.org/obo/IDO_0100879>; rdf:rest ?n4 .
 ?n4 rdf:first ?n5 .
 ?n5 owl:onProperty <http://purl.obolibrary.org/obo/BFO_0000054>; owl:someValuesFrom
<http://purl.obolibrary.org/obo/IDO_0100832> .
```

```
 ?s rdfs:subClassOf ?n .
 }
}
FILTER REGEX(str(?p), "PR") .
}

##############################
# Result:  (as returned by the server on 3/24/2015 12:20pm)
```

(269 rows)

s	l
http://purl.obolibrary.org/obo/IDO_0110000	B. suis 1330 rbsK mutant
http://purl.obolibrary.org/obo/IDO_0110001	B. suis 1330 mgtB mutant
http://purl.obolibrary.org/obo/IDO_0110002	B. suis 1330 nodV mutant
http://purl.obolibrary.org/obo/IDO_0110006	B. suis 1330 virB9 mutant
http://purl.obolibrary.org/obo/IDO_0110009	B. suis 1330 virB5 mutant
http://purl.obolibrary.org/obo/IDO_0110010	B. suis 1330 virB4 mutant
http://purl.obolibrary.org/obo/IDO_0110012	B. suis 1330 virB2 mutant
http://purl.obolibrary.org/obo/IDO_0110013	B. suis 1330 virB1 mutant
http://purl.obolibrary.org/obo/IDO_0110014	B. suis 1330 hemH mutant
http://purl.obolibrary.org/obo/IDO_0110017	B. suis 1330 vjbR mutant
http://purl.obolibrary.org/obo/IDO_0110018	B. suis 1330 gtrB mutant
http://purl.obolibrary.org/obo/IDO_0110019	B. suis 1330 deoR mutant
http://purl.obolibrary.org/obo/IDO_0110021	B. suis 1330 gntR mutant
http://purl.obolibrary.org/obo/IDO_0110022	B. suis 1330 caiB mutant
http://purl.obolibrary.org/obo/IDO_0110024	B. suis 1330 norD mutant
http://purl.obolibrary.org/obo/IDO_0110025	B. suis 1330 narG mutant
http://purl.obolibrary.org/obo/IDO_0110027	B. suis 1330 wbpW mutant
http://purl.obolibrary.org/obo/IDO_0110028	B. suis 1330 manB mutant
http://purl.obolibrary.org/obo/IDO_0110030	B. suis 1330 xfp mutant
http://purl.obolibrary.org/obo/IDO_0110031	B. suis 1330 glpK mutant
http://purl.obolibrary.org/obo/IDO_0110034	B. suis 1330 cydC mutant
http://purl.obolibrary.org/obo/IDO_0110036	B. suis 1330 rbsC mutant
http://purl.obolibrary.org/obo/IDO_0110038	B. suis 1330 aidB mutant
http://purl.obolibrary.org/obo/IDO_0110044	B. suis 1330 fbpA mutant
http://purl.obolibrary.org/obo/IDO_0110045	B. suis 1330 sodC mutant
http://purl.obolibrary.org/obo/IDO_0110048	B. suis 1330 gcvT mutant
http://purl.obolibrary.org/obo/IDO_0110050	B. suis 1330 zwf mutant
http://purl.obolibrary.org/obo/IDO_0110051	B. suis 1330 nikA mutant
http://purl.obolibrary.org/obo/IDO_0110052	B. suis 1330 galcD mutant
http://purl.obolibrary.org/obo/IDO_0110053	B. suis 1330 eryB mutant
http://purl.obolibrary.org/obo/IDO_0110057	B. suis 1330 dacF mutant
http://purl.obolibrary.org/obo/IDO_0110058	B. suis 1330 cobW mutant
http://purl.obolibrary.org/obo/IDO_0110059	B. suis 1330 dppA mutant

http://purl.obolibrary.org/obo/IDO_0110060	B. suis 1330 znuA mutant
http://purl.obolibrary.org/obo/IDO_0110061	B. suis 1330 znuC mutant
http://purl.obolibrary.org/obo/IDO_0110065	B. suis 1330 rpsA mutant
http://purl.obolibrary.org/obo/IDO_0110066	B. suis 1330 pheA mutant
http://purl.obolibrary.org/obo/IDO_0110068	B. suis 1330 ilvD mutant
http://purl.obolibrary.org/obo/IDO_0110070	B. suis 1330 glnD mutant
http://purl.obolibrary.org/obo/IDO_0110071	B. suis 1330 cysI mutant
http://purl.obolibrary.org/obo/IDO_0110072	B. suis 1330 metH mutant
http://purl.obolibrary.org/obo/IDO_0110073	B. suis 1330 malK mutant
http://purl.obolibrary.org/obo/IDO_0110074	B. suis 1330 hisD mutant
http://purl.obolibrary.org/obo/IDO_0110075	B. suis 1330 pgi mutant
http://purl.obolibrary.org/obo/IDO_0110077	B. suis 1330 vsrB mutant
http://purl.obolibrary.org/obo/IDO_0110078	B. suis 1330 bacA mutant
http://purl.obolibrary.org/obo/IDO_0110079	B. suis 1330 purD mutant
http://purl.obolibrary.org/obo/IDO_0110080	B. suis 1330 aroC mutant
http://purl.obolibrary.org/obo/IDO_0110082	B. suis 1330 purF mutant
http://purl.obolibrary.org/obo/IDO_0110084	B. suis 1330 thrC mutant
http://purl.obolibrary.org/obo/IDO_0110085	B. suis 1330 dsbA mutant
http://purl.obolibrary.org/obo/IDO_0110086	B. suis 1330 wbpL mutant
http://purl.obolibrary.org/obo/IDO_0110088	B. suis 1330 rfbD mutant
http://purl.obolibrary.org/obo/IDO_0110089	B. suis 1330 perA mutant
http://purl.obolibrary.org/obo/IDO_0110092	B. suis 1330 pmm mutant
http://purl.obolibrary.org/obo/IDO_0110093	B. suis 1330 wbpZ mutant
http://purl.obolibrary.org/obo/IDO_0110094	B. suis 1330 feuP mutant
http://purl.obolibrary.org/obo/IDO_0110095	B. suis 1330 feuQ mutant
http://purl.obolibrary.org/obo/IDO_0110096	B. suis 1330 htrA mutant
http://purl.obolibrary.org/obo/IDO_0110097	B. suis 1330 lpsA mutant
http://purl.obolibrary.org/obo/IDO_0110099	B. suis 1330 spotT mutant
http://purl.obolibrary.org/obo/IDO_0110104	B. suis 1330 glyA mutant
http://purl.obolibrary.org/obo/IDO_0110105	B. suis 1330 purL mutant
http://purl.obolibrary.org/obo/IDO_0110107	B. suis 1330 dsbA mutant
http://purl.obolibrary.org/obo/IDO_0110108	B. suis 1330 amiC mutant
http://purl.obolibrary.org/obo/IDO_0110109	B. suis 1330 nifS mutant
http://purl.obolibrary.org/obo/IDO_0110110	B. suis 1330 dsbA mutant
http://purl.obolibrary.org/obo/IDO_0110112	B. suis 1330 wbdA mutant
http://purl.obolibrary.org/obo/IDO_0110113	B. suis 1330 glnA mutant
http://purl.obolibrary.org/obo/IDO_0110114	B. suis 1330 cysK mutant
http://purl.obolibrary.org/obo/IDO_0110115	B. suis 1330 caiB mutant
http://purl.obolibrary.org/obo/IDO_0110116	B. suis 1330 uvrA mutant
http://purl.obolibrary.org/obo/IDO_0110118	B. suis 1330 hfq mutant
http://purl.obolibrary.org/obo/IDO_0110121	B. suis 1330 ppiD mutant
http://purl.obolibrary.org/obo/IDO_0110122	B. suis 1330 uppS mutant
http://purl.obolibrary.org/obo/IDO_0110125	B. suis 1330 gloA mutant

http://purl.obolibrary.org/obo/IDO_0110127	B. suis 1330 aspB mutant
http://purl.obolibrary.org/obo/IDO_0110129	B. suis 1330 miaA mutant
http://purl.obolibrary.org/obo/IDO_0110130	B. suis 1330 serB mutant
http://purl.obolibrary.org/obo/IDO_0110131	B. suis 1330 pncA mutant
http://purl.obolibrary.org/obo/IDO_0110132	B. suis 1330 pncA mutant
http://purl.obolibrary.org/obo/IDO_0110134	B. suis 1330 lysR mutant
http://purl.obolibrary.org/obo/IDO_0110135	B. suis 1330 pth mutant
http://purl.obolibrary.org/obo/IDO_0110136	B. suis 1330 leuA mutant
http://purl.obolibrary.org/obo/IDO_0110137	B. suis 1330 dsbB mutant
http://purl.obolibrary.org/obo/IDO_0110138	B. suis 1330 alkA mutant
http://purl.obolibrary.org/obo/IDO_0110139	B. suis 1330 macA mutant
http://purl.obolibrary.org/obo/IDO_0110140	B. suis 1330 dut mutant
http://purl.obolibrary.org/obo/IDO_0110141	B. suis 1330 ansC mutant
http://purl.obolibrary.org/obo/IDO_0110142	B. suis 1330 purE mutant
http://purl.obolibrary.org/obo/IDO_0110143	B. suis 1330 pyc mutant
http://purl.obolibrary.org/obo/IDO_0110145	B. suis 1330 purH mutant
http://purl.obolibrary.org/obo/IDO_0110146	B. suis 1330 leuC mutant
http://purl.obolibrary.org/obo/IDO_0110147	B. suis 1330 rplS mutant
http://purl.obolibrary.org/obo/IDO_0110149	B. suis 1330 lysA mutant
http://purl.obolibrary.org/obo/IDO_0110150	B. suis 1330 hpt mutant
http://purl.obolibrary.org/obo/IDO_0110152	B. suis 1330 hisF mutant
http://purl.obolibrary.org/obo/IDO_0110153	B. suis 1330 bvrR mutant
http://purl.obolibrary.org/obo/IDO_0110154	B. suis 1330 bvrS mutant
http://purl.obolibrary.org/obo/IDO_0110155	B. suis 1330 dnaK mutant
http://purl.obolibrary.org/obo/IDO_0110156	B. suis 1330 pmtA mutant
http://purl.obolibrary.org/obo/IDO_0110158	B. melitensis 16M hpt mutant
http://purl.obolibrary.org/obo/IDO_0110159	B. melitensis 16M lysA mutant
http://purl.obolibrary.org/obo/IDO_0110160	B. melitensis 16M BMEI0085 mutant
http://purl.obolibrary.org/obo/IDO_0110162	B. melitensis 16M rplS mutant
http://purl.obolibrary.org/obo/IDO_0110163	B. melitensis 16M leuC mutant
http://purl.obolibrary.org/obo/IDO_0110165	B. melitensis 16M pstP mutant
http://purl.obolibrary.org/obo/IDO_0110166	B. melitensis 16M purH mutant
http://purl.obolibrary.org/obo/IDO_0110168	B. melitensis 16M pyc mutant
http://purl.obolibrary.org/obo/IDO_0110170	B. melitensis 16M mgps mutant
http://purl.obolibrary.org/obo/IDO_0110171	B. melitensis 16M purE mutant
http://purl.obolibrary.org/obo/IDO_0110174	B. melitensis 16M ansC mutant
http://purl.obolibrary.org/obo/IDO_0110175	B. melitensis 16M dut mutant
http://purl.obolibrary.org/obo/IDO_0110176	B. melitensis 16M macA mutant
http://purl.obolibrary.org/obo/IDO_0110177	B. melitensis 16M alkA mutant
http://purl.obolibrary.org/obo/IDO_0110178	B. melitensis 16M dsbB mutant
http://purl.obolibrary.org/obo/IDO_0110180	B. melitensis 16M dppA mutant
http://purl.obolibrary.org/obo/IDO_0110181	B. melitensis 16M leuA mutant
http://purl.obolibrary.org/obo/IDO_0110182	B. melitensis 16M BMEI0455 mutant

http://purl.obolibrary.org/obo/IDO_0110183	B. melitensis 16M pth mutant
http://purl.obolibrary.org/obo/IDO_0110184	B. melitensis 16M lpsB mutant
http://purl.obolibrary.org/obo/IDO_0110186	B. melitensis 16M lysR mutant
http://purl.obolibrary.org/obo/IDO_0110188	B. melitensis 16M carAB mutant
http://purl.obolibrary.org/obo/IDO_0110189	B. melitensis 16M pncA mutant
http://purl.obolibrary.org/obo/IDO_0110190	B. melitensis 16M bicA mutant
http://purl.obolibrary.org/obo/IDO_0110191	B. melitensis 16M serB mutant
http://purl.obolibrary.org/obo/IDO_0110192	B. melitensis 16M miaA mutant
http://purl.obolibrary.org/obo/IDO_0110193	B. melitensis 16M ilvI mutant
http://purl.obolibrary.org/obo/IDO_0110195	B. melitensis 16M aspB mutant
http://purl.obolibrary.org/obo/IDO_0110196	B. melitensis 16M BMEI0671 mutant
http://purl.obolibrary.org/obo/IDO_0110198	B. melitensis 16M thrA mutant
http://purl.obolibrary.org/obo/IDO_0110199	B. melitensis 16M gloA mutant
http://purl.obolibrary.org/obo/IDO_0110202	B. melitensis 16M uppS mutant
http://purl.obolibrary.org/obo/IDO_0110203	B. melitensis 16M ppiD mutant
http://purl.obolibrary.org/obo/IDO_0110205	B. melitensis 16M hfq mutant
http://purl.obolibrary.org/obo/IDO_0110207	B. melitensis 16M uvrA mutant
http://purl.obolibrary.org/obo/IDO_0110209	B. melitensis 16M caiB mutant
http://purl.obolibrary.org/obo/IDO_0110210	B. melitensis 16M cysK mutant
http://purl.obolibrary.org/obo/IDO_0110211	B. melitensis 16M glnA mutant
http://purl.obolibrary.org/obo/IDO_0110212	B. melitensis 16M wbdA mutant
http://purl.obolibrary.org/obo/IDO_0110214	B. melitensis 16M dsbA mutant
http://purl.obolibrary.org/obo/IDO_0110215	B. melitensis 16M nifS mutant
http://purl.obolibrary.org/obo/IDO_0110216	B. melitensis 16M amiC mutant
http://purl.obolibrary.org/obo/IDO_0110217	B. melitensis 16M dsbA mutant
http://purl.obolibrary.org/obo/IDO_0110220	B. melitensis 16M cbbE mutant
http://purl.obolibrary.org/obo/IDO_0110221	B. melitensis 16M purL mutant
http://purl.obolibrary.org/obo/IDO_0110222	B. melitensis 16M glyA mutant
http://purl.obolibrary.org/obo/IDO_0110224	B. melitensis 16M galE mutant
http://purl.obolibrary.org/obo/IDO_0110229	B. melitensis 16M pyrC mutant
http://purl.obolibrary.org/obo/IDO_0110230	B. melitensis 16M spotT mutant
http://purl.obolibrary.org/obo/IDO_0110231	B. melitensis 16M lpsA mutant
http://purl.obolibrary.org/obo/IDO_0110232	B. melitensis 16M htrA mutant
http://purl.obolibrary.org/obo/IDO_0110233	B. melitensis 16M feuQ mutant
http://purl.obolibrary.org/obo/IDO_0110234	B. melitensis 16M feuP mutant
http://purl.obolibrary.org/obo/IDO_0110235	B. melitensis 16M BMEI1339 mutant
http://purl.obolibrary.org/obo/IDO_0110237	B. melitensis 16M wbpZ mutant
http://purl.obolibrary.org/obo/IDO_0110238	B. melitensis 16M pmm mutant
http://purl.obolibrary.org/obo/IDO_0110240	B. melitensis 16M rfbD mutant
http://purl.obolibrary.org/obo/IDO_0110241	B. melitensis 16M perA mutant
http://purl.obolibrary.org/obo/IDO_0110242	B. melitensis 16M wbpL mutant
http://purl.obolibrary.org/obo/IDO_0110243	B. melitensis 16M dsbA mutant
http://purl.obolibrary.org/obo/IDO_0110244	B. melitensis 16M BMEI1443 mutant

http://purl.obolibrary.org/obo/IDO_0110245	B. melitensis 16M BMEI1448 mutant
http://purl.obolibrary.org/obo/IDO_0110246	B. melitensis 16M thrC mutant
http://purl.obolibrary.org/obo/IDO_0110248	B. melitensis 16M purF mutant
http://purl.obolibrary.org/obo/IDO_0110250	B. melitensis 16M aroC mutant
http://purl.obolibrary.org/obo/IDO_0110251	B. melitensis 16M purD mutant
http://purl.obolibrary.org/obo/IDO_0110252	B. melitensis 16M BMEI1531 mutant
http://purl.obolibrary.org/obo/IDO_0110253	B. melitensis 16M bacA mutant
http://purl.obolibrary.org/obo/IDO_0110255	B. melitensis 16M vsrB mutant
http://purl.obolibrary.org/obo/IDO_0110257	B. melitensis 16M pgi mutant
http://purl.obolibrary.org/obo/IDO_0110258	B. melitensis 16M BMEI1658 mutant
http://purl.obolibrary.org/obo/IDO_0110259	B. melitensis 16M hisD mutant
http://purl.obolibrary.org/obo/IDO_0110260	B. melitensis 16M malK mutant
http://purl.obolibrary.org/obo/IDO_0110262	B. melitensis 16M metH mutant
http://purl.obolibrary.org/obo/IDO_0110263	B. melitensis 16M cysI mutant
http://purl.obolibrary.org/obo/IDO_0110264	B. melitensis 16M glnL mutant
http://purl.obolibrary.org/obo/IDO_0110265	B. melitensis 16M glnD mutant
http://purl.obolibrary.org/obo/IDO_0110266	B. melitensis 16M BMEI1809 mutant
http://purl.obolibrary.org/obo/IDO_0110268	B. melitensis 16M BMEI1844 mutant
http://purl.obolibrary.org/obo/IDO_0110269	B. melitensis 16M ilvD mutant
http://purl.obolibrary.org/obo/IDO_0110270	B. melitensis 16M cysY mutant
http://purl.obolibrary.org/obo/IDO_0110271	B. melitensis 16M BMEI1859 mutant
http://purl.obolibrary.org/obo/IDO_0110272	B. melitensis 16M BMEI1879 mutant
http://purl.obolibrary.org/obo/IDO_0110274	B. melitensis 16M BMEI1902 mutant
http://purl.obolibrary.org/obo/IDO_0110276	B. melitensis 16M rpsA mutant
http://purl.obolibrary.org/obo/IDO_0110278	B. melitensis 16M dnaK mutant
http://purl.obolibrary.org/obo/IDO_0110279	B. melitensis 16M bvrS mutant
http://purl.obolibrary.org/obo/IDO_0110280	B. melitensis 16M bvrR mutant
http://purl.obolibrary.org/obo/IDO_0110281	B. melitensis 16M hisF mutant
http://purl.obolibrary.org/obo/IDO_0110283	B. melitensis 16M hemH mutant
http://purl.obolibrary.org/obo/IDO_0110284	B. melitensis 16M virb1 mutant
http://purl.obolibrary.org/obo/IDO_0110285	B. melitensis 16M virB2 mutant
http://purl.obolibrary.org/obo/IDO_0110287	B. melitensis 16M virb4 mutant
http://purl.obolibrary.org/obo/IDO_0110288	B. melitensis 16M virB5 mutant
http://purl.obolibrary.org/obo/IDO_0110291	B. melitensis 16M virb9 mutant
http://purl.obolibrary.org/obo/IDO_0110293	B. melitensis 16M virb11 mutant
http://purl.obolibrary.org/obo/IDO_0110296	B. melitensis 16M nodV mutant
http://purl.obolibrary.org/obo/IDO_0110297	B. melitensis 16M mgtB mutant
http://purl.obolibrary.org/obo/IDO_0110299	B. melitensis 16M rbsK mutant
http://purl.obolibrary.org/obo/IDO_0110301	B. melitensis 16M BMEII0128 mutant
http://purl.obolibrary.org/obo/IDO_0110303	B. melitensis 16M fliC mutant
http://purl.obolibrary.org/obo/IDO_0110309	B. melitensis 16M znuC mutant
http://purl.obolibrary.org/obo/IDO_0110310	B. melitensis 16M znuA mutant
http://purl.obolibrary.org/obo/IDO_0110311	B. melitensis 16M BMEII0274 mutant

http://purl.obolibrary.org/obo/IDO_0110313	B. melitensis 16M cobW mutant
http://purl.obolibrary.org/obo/IDO_0110314	B. melitensis 16M BMEII0318 mutant
http://purl.obolibrary.org/obo/IDO_0110315	B. melitensis 16M BMEII0336 mutant
http://purl.obolibrary.org/obo/IDO_0110316	B. melitensis 16M dacF mutant
http://purl.obolibrary.org/obo/IDO_0110321	B. melitensis 16M eryB mutant
http://purl.obolibrary.org/obo/IDO_0110322	B. melitensis 16M gntR1 mutant
http://purl.obolibrary.org/obo/IDO_0110323	B. melitensis 16M galcD mutant
http://purl.obolibrary.org/obo/IDO_0110324	B. melitensis 16M nikA mutant
http://purl.obolibrary.org/obo/IDO_0110325	B. melitensis 16M zwf mutant
http://purl.obolibrary.org/obo/IDO_0110329	B. melitensis 16M sodC mutant
http://purl.obolibrary.org/obo/IDO_0110333	B. melitensis 16M BMEII0626 mutant
http://purl.obolibrary.org/obo/IDO_0110335	B. melitensis 16M pyrC mutant
http://purl.obolibrary.org/obo/IDO_0110337	B. melitensis 16M aidB mutant
http://purl.obolibrary.org/obo/IDO_0110338	B. melitensis 16M rbsC mutant
http://purl.obolibrary.org/obo/IDO_0110340	B. melitensis 16M cydC mutant
http://purl.obolibrary.org/obo/IDO_0110343	B. melitensis 16M glpK mutant
http://purl.obolibrary.org/obo/IDO_0110344	B. melitensis 16M xfp mutant
http://purl.obolibrary.org/obo/IDO_0110345	B. melitensis 16M manB mutant
http://purl.obolibrary.org/obo/IDO_0110346	B. melitensis 16M wbpW mutant
http://purl.obolibrary.org/obo/IDO_0110350	B. melitensis 16M BMEII0935 mutant
http://purl.obolibrary.org/obo/IDO_0110351	B. melitensis 16M narG mutant
http://purl.obolibrary.org/obo/IDO_0110354	B. melitensis 16M BMEII1045 mutant
http://purl.obolibrary.org/obo/IDO_0110356	B. melitensis 16M gntR mutant
http://purl.obolibrary.org/obo/IDO_0110358	B. melitensis 16M deoR mutant
http://purl.obolibrary.org/obo/IDO_0110359	B. melitensis 16M gtrB mutant
http://purl.obolibrary.org/obo/IDO_0110360	B. melitensis 16M vjbR mutant
http://purl.obolibrary.org/obo/IDO_0110362	B. abortus 2308 metH mutant
http://purl.obolibrary.org/obo/IDO_0110363	B. abortus 2308 pgi mutant
http://purl.obolibrary.org/obo/IDO_0110365	B. abortus 2308 uvrA mutant
http://purl.obolibrary.org/obo/IDO_0110366	B. abortus 2308 miaA mutant
http://purl.obolibrary.org/obo/IDO_0110367	B. abortus 2308 aroC mutant
http://purl.obolibrary.org/obo/IDO_0110369	B. abortus 2308 bacA mutant
http://purl.obolibrary.org/obo/IDO_0110370	B. abortus 2308 cysI mutant
http://purl.obolibrary.org/obo/IDO_0110371	B. abortus 2308 dut mutant
http://purl.obolibrary.org/obo/IDO_0110372	B. abortus 2308 glnA mutant
http://purl.obolibrary.org/obo/IDO_0110373	B. abortus 2308 gloA mutant
http://purl.obolibrary.org/obo/IDO_0110374	B. abortus 2308 glyA mutant
http://purl.obolibrary.org/obo/IDO_0110376	B. abortus 2308 hfq mutant
http://purl.obolibrary.org/obo/IDO_0110378	B. abortus 2308 hisD mutant
http://purl.obolibrary.org/obo/IDO_0110379	B. abortus 2308 hisF mutant
http://purl.obolibrary.org/obo/IDO_0110380	B. abortus 2308 hpt mutant
http://purl.obolibrary.org/obo/IDO_0110381	B. abortus 2308 ilvD mutant
http://purl.obolibrary.org/obo/IDO_0110382	B. abortus 2308 leuA mutant

http://purl.obolibrary.org/obo/IDO_0110383	B. abortus 2308 leuC mutant
http://purl.obolibrary.org/obo/IDO_0110384	B. abortus 2308 lysA mutant
http://purl.obolibrary.org/obo/IDO_0110390	B. abortus 2308 pheA mutant
http://purl.obolibrary.org/obo/IDO_0110391	B. abortus 2308 pth mutant
http://purl.obolibrary.org/obo/IDO_0110392	B. abortus 2308 purD mutant
http://purl.obolibrary.org/obo/IDO_0110393	B. abortus 2308 purF mutant
http://purl.obolibrary.org/obo/IDO_0110394	B. abortus 2308 purH mutant
http://purl.obolibrary.org/obo/IDO_0110397	B. abortus 2308 pyc mutant
http://purl.obolibrary.org/obo/IDO_0110400	B. abortus 2308 rfbD mutant
http://purl.obolibrary.org/obo/IDO_0110401	B. abortus 2308 rplS mutant
http://purl.obolibrary.org/obo/IDO_0110403	B. abortus 2308 rpsA mutant
http://purl.obolibrary.org/obo/IDO_0110408	B. abortus 2308 znuA mutant
http://purl.obolibrary.org/obo/IDO_0110409	B. abortus 2308 znuC mutant
http://purl.obolibrary.org/obo/IDO_0110412	B. abortus 2308 sodC mutant
http://purl.obolibrary.org/obo/IDO_0110415	B. abortus 2308 narG mutant
http://purl.obolibrary.org/obo/IDO_0110416	B. abortus 2308 virB5 mutant
http://purl.obolibrary.org/obo/IDO_0110418	B. abortus 2308 virB2 mutant
http://purl.obolibrary.org/obo/IDO_0110424	B. abortus 2308 gcvT mutant
http://purl.obolibrary.org/obo/IDO_0110427	B. abortus 2308 hemH mutant
http://purl.obolibrary.org/obo/IDO_0110428	B. melitensis 16M BMEI0066 mutant
http://purl.obolibrary.org/obo/IDO_0110431	B. abortus 2308 pgk mutant

############################
