# Supplementary material for: Ontology-based representation and analysis of host-Brucella interactions
Source: J Biomed Semantics. 2015 Oct 5;6:37. doi: 10.1186/s13326-015-0036-y (PMC4594885; doi:10.1186/s13326-015-0036-y)
Supplement: Additional file 4: — SPARQL query of IDOBRU for Brucella protein virulence factors important for the intracellular replication of Brucella inside macrophages. (PDF 198 kb) [file 13326_2015_36_MOESM4_ESM.pdf]

Supplemental file – SPARQL queries of *Brucella* Protein Virulence Factors important for *Brucella* intracellular replication in macrophages

This supplemental file includes two scripts, both related to the queries of the protein virulence factors that are related with *Brucella* intracellular replication in macrophages.

- SPARQL query 2: SPARQL query of the total of all the protein virulence factors that are related with *Brucella* intracellular replication in macrophages.
- SPARQL query 2.1: SPARQL query of the list of all the protein virulence factors that are related with *Brucella* intracellular replication in macrophages.

```
##############################
#this query was running on http://sparql.hegroup.org/sparql
# Date: on 3/24/2015
# IDOBRU version: 86
#
###############################


#SPARQL query2: the total of all the protein virulence factors that are related with Brucella intracellular replication in macrophages.

# BFO_0000051 has part;
# IDO_0101168 agent_in_compromised_process;
# IDO_0100612 Brucella intracellular replication in macrophage

prefix rdf: <http://www.w3.org/1999/02/22-rdf-syntax-ns#>
prefix owl: <http://www.w3.org/2002/07/owl#>

SELECT (count(distinct ?s) as ?count)
from <http://purl.obolibrary.org/obo/merged/IDOBRU>

WHERE {
 ?n2 rdfs:subClassOf ?s1 .
 ?s1 owl:complementOf ?s2 .
?s2  owl:onProperty <http://purl.obolibrary.org/obo/BFO_0000051>; owl:someValuesFrom ?s .
 ?s rdfs:label ?l .
 {
 SELECT ?n2
 WHERE
 {
 ?n rdfs:subClassOf ?n1 .
 ?n1 owl:onProperty <http://purl.obolibrary.org/obo/IDO_0101168>; owl:someValuesFrom
<http://purl.obolibrary.org/obo/IDO_0100612>.
 ?n2 rdfs:subClassOf ?n .
 }
 }
 FILTER REGEX(str(?s), "PR") .
```

```
}

#############################
#Result: (as returned by the server on 3/24/2015 12:31am)
count
81
#############################



#SPARQL query2.1: the list of all the protein virulence factors that are related with Brucella intracellular
replication in macrophages.

# IDO_0100738 lacks_part;
# IDO_0101168 agent_in_compromised_process;
# IDO_0100612 Brucella intracellular replication in macrophage

prefix rdf: <http://www.w3.org/1999/02/22-rdf-syntax-ns#>
prefix owl: <http://www.w3.org/2002/07/owl#>

SELECT ?s ?l
from <http://purl.obolibrary.org/obo/merged/IDOBRU>

WHERE {
 ?n2 rdfs:subClassOf ?s1 .
 ?s1 owl:complementOf ?s2 .
 ?s2 owl:onProperty <http://purl.obolibrary.org/obo/BFO_0000051>; owl:someValuesFrom ?s .
 ?s rdfs:label ?l .
 {
 SELECT ?n2
 WHERE
 {
 ?n rdfs:subClassOf ?n1 .
 ?n1 owl:onProperty <http://purl.obolibrary.org/obo/IDO_0101168>; owl:someValuesFrom
<http://purl.obolibrary.org/obo/IDO_0100612>.
 ?n2 rdfs:subClassOf ?n .
 }
 }
 FILTER REGEX(str(?s), "PR") .

}


#########################
#Result:(as returned by the server on 3/24/2015 12:36am)
```

s	l
http://purl.obolibrary.org/obo/PR_Q9RPY4	type IV secretion system protein VirB1
http://purl.obolibrary.org/obo/PR_Q8YDZ5	attachment mediating protein VIRB1-like protein
http://purl.obolibrary.org/obo/PR_Q9RPY3	VirB2
http://purl.obolibrary.org/obo/PR_Q2YIT6	type IV secretion system protein VirB2 KO: K03197 type IV secretion system protein VirB2
http://purl.obolibrary.org/obo/PR_Q7CEG0	type IV secretion system protein VirB2
http://purl.obolibrary.org/obo/PR_P52558	PHOSPHORIBOSYLAMINOIMIDAZOLE CARBOXYLASE CATALYTIC SUBUNIT
http://purl.obolibrary.org/obo/PR_Q8G183	phosphoribosylformylglycinamidine synthase II
http://purl.obolibrary.org/obo/PR_Q8YGN1	phosphoribosylformylglycinamidine synthase II
http://purl.obolibrary.org/obo/PR_Q2YMD3	phosphoribosylamine--glycine ligase
http://purl.obolibrary.org/obo/PR_Q8G2B1	phosphoribosylamine--glycine ligase
http://purl.obolibrary.org/obo/PR_Q8YFK1	phosphoribosylamine--glycine ligase
http://purl.obolibrary.org/obo/PR_Q8G353	dihydroxy-acid dehydratase
http://purl.obolibrary.org/obo/PR_Q2YNW9	dihydroxy-acid dehydratase
http://purl.obolibrary.org/obo/PR_Q8YEN0	dihydroxy-acid dehydratase
http://purl.obolibrary.org/obo/PR_Q8FZL6	pyrazinamidase/nicotinamidase
http://purl.obolibrary.org/obo/PR_Q8YIA0	INTEGRAL MEMBRANE PROTEIN
http://purl.obolibrary.org/obo/PR_Q8FZL5	hypothetical protein
http://purl.obolibrary.org/obo/PR_P0A3G8	RNA-binding protein Hfq
http://purl.obolibrary.org/obo/PR_P0A3G7	RNA-binding protein Hfq
http://purl.obolibrary.org/obo/PR_Q2YPW9	RNA-binding protein Hfq
http://purl.obolibrary.org/obo/PR_Q2YN95	serine hydroxymethyltransferase
http://purl.obolibrary.org/obo/PR_Q8YGG7	SERINE HYDROXYMETHYLTRANSFERASE
http://purl.obolibrary.org/obo/PR_Q8G1F1	serine hydroxymethyltransferase
http://purl.obolibrary.org/obo/PR_Q8YF59	histidinol dehydrogenase
http://purl.obolibrary.org/obo/PR_Q8G2R2	histidinol dehydrogenase
http://purl.obolibrary.org/obo/PR_Q2YPB8	histidinol dehydrogenase
http://purl.obolibrary.org/obo/PR_Q8YIJ3	2-isopropylmalate synthase
http://purl.obolibrary.org/obo/PR_Q2YRT1	2-isopropylmalate synthase
http://purl.obolibrary.org/obo/PR_Q8FZC4	2-isopropylmalate synthase
http://purl.obolibrary.org/obo/PR_Q8YJC9	isopropylmalate isomerase large subunit
http://purl.obolibrary.org/obo/PR_Q8FYG9	isopropylmalate isomerase large subunit
http://purl.obolibrary.org/obo/PR_Q2YLP7	isopropylmalate isomerase large subunit

http://purl.obolibrary.org/obo/PR_Q8FYA2	diaminopimelate decarboxylase
http://purl.obolibrary.org/obo/PR_Q2YR85	Orn/DAP/Arg decarboxylase, family 2:ATP/GTP-binding site motif A (P-loop):Diaminopimelate decarboxylase
http://purl.obolibrary.org/obo/PR_Q8YJJ9	DIAMINOPIMELATE DECARBOXYLASE
http://purl.obolibrary.org/obo/PR_Q8G0D9	undecaprenyl diphosphate synthase
http://purl.obolibrary.org/obo/PR_Q8YHH3	UNDECAPRENYL PYROPHOSPHATE SYNTHETASE
http://purl.obolibrary.org/obo/PR_Q8CY40	tRNA delta(2)-isopentenylpyrophosphate transferase
http://purl.obolibrary.org/obo/PR_Q2YS31	tRNA delta(2)-isopentenylpyrophosphate transferase
http://purl.obolibrary.org/obo/PR_Q8YI29	tRNA delta(2)-isopentenylpyrophosphate transferase
http://purl.obolibrary.org/obo/PR_Q8FV62	cobalamin synthesis protein/P47K family protein
http://purl.obolibrary.org/obo/PR_Q8YD69	LOW AFFINITY ZINC TRANSPORT MEMBRANE PROTEIN
http://purl.obolibrary.org/obo/PR_Q8G2F1	transport protein
http://purl.obolibrary.org/obo/PR_Q8YFG9	transport protein
http://purl.obolibrary.org/obo/PR_Q2YMA1	transport protein
http://purl.obolibrary.org/obo/PR_Q8YF86	glucose-6-phosphate isomerase
http://purl.obolibrary.org/obo/PR_Q8G2N3	glucose-6-phosphate isomerase
http://purl.obolibrary.org/obo/PR_Q2YPF3	glucose-6-phosphate isomerase
http://purl.obolibrary.org/obo/PR_Q7CEG3	type IV secretion system protein VirB8
http://purl.obolibrary.org/obo/PR_Q2YJ78	VirB8
http://purl.obolibrary.org/obo/PR_Q9RPX7	VirB8
http://purl.obolibrary.org/obo/PR_Q9RPX6	type IV secretion system protein VirB9
http://purl.obolibrary.org/obo/PR_Q8YDZ1	CHANNEL PROTEIN VIRB9 HOMOLOG
http://purl.obolibrary.org/obo/PR_Q8YDZ0	channel protein VIRB10-like protein
http://purl.obolibrary.org/obo/PR_Q9RPX5	type IV secretion system protein VirB10
http://purl.obolibrary.org/obo/PR_Q9RPY1	type IV secretion system protein VirB4
http://purl.obolibrary.org/obo/PR_Q8FWZ3	respiratory nitrate reductase, alpha subunit
http://purl.obolibrary.org/obo/PR_Q8YBF0	NITRATE REDUCTASE ALPHA CHAIN
http://purl.obolibrary.org/obo/PR_Q2YJY4	Nitrate reductase, alpha subunit:Prokaryotic molybdopterin oxidoreductase:Molybdopterin oxidoreductase:Molydopterin dinucleot...
http://purl.obolibrary.org/obo/PR_Q8FW88	phosphatidylcholine synthase

http://purl.obolibrary.org/obo/PR_Q8YH25	GLUTAMINE SYNTHETASE
http://purl.obolibrary.org/obo/PR_Q2YQ67	Glutamine synthetase class-I, adenylation site:Glutamine synthetase type I:Glutamine synthetase, catalytic domain:Glutamine s...
http://purl.obolibrary.org/obo/PR_Q8G0T3	glutamine synthetase, type I
http://purl.obolibrary.org/obo/PR_Q8YI30	PHOSPHOSERINE PHOSPHATASE
http://purl.obolibrary.org/obo/PR_Q8FZT1	phosphoserine phosphatase
http://purl.obolibrary.org/obo/PR_P64005	deoxyuridine 5'-triphosphate nucleotidohydrolase
http://purl.obolibrary.org/obo/PR_Q2YRG4	deoxyuridine 5'-triphosphate nucleotidohydrolase
http://purl.obolibrary.org/obo/PR_Q8FZ27	transcriptional regulator, AsnC family
http://purl.obolibrary.org/obo/PR_P64004	deoxyuridine 5'-triphosphate nucleotidohydrolase
http://purl.obolibrary.org/obo/PR_Q8YE37	imidazole glycerol phosphate synthase subunit HisF
http://purl.obolibrary.org/obo/PR_Q2YQY8	imidazole glycerol phosphate synthase subunit HisF
http://purl.obolibrary.org/obo/PR_Q8FY07	imidazole glycerol phosphate synthase subunit HisF
http://purl.obolibrary.org/obo/PR_Q8FY04	DNA-binding response regulator BvrR, putative
http://purl.obolibrary.org/obo/PR_Q8YE42	TRANSCRIPTIONAL REGULATORY PROTEIN CHVI
http://purl.obolibrary.org/obo/PR_Q8YE76	molecular chaperone DnaK
http://purl.obolibrary.org/obo/PR_Q8FXX2	molecular chaperone DnaK
http://purl.obolibrary.org/obo/PR_Q8FXX0	phospholipid N-methyltransferase
http://purl.obolibrary.org/obo/PR_Q8YE43	SENSOR PROTEIN CHVG
http://purl.obolibrary.org/obo/PR_Q8FY03	sensor histidine kinase BvrS, putative
http://purl.obolibrary.org/obo/PR_Q8YDZ4	ATPASE VIRB4 HOMOLOG
http://purl.obolibrary.org/obo/PR_Q8YJL6	two component response regulator
